# *Drosophila* Trus, the orthologue of mammalian PDCD2L, is required for proper cell proliferation, larval developmental timing, and oogenesis

**DOI:** 10.1371/journal.pgen.1011469

**Published:** 2025-06-27

**Authors:** Saeko Takada, Bonnie J. Bolkan, MaryJane O’Connor, Michael Goldberg, Michael B. O’Connor

**Affiliations:** 1 Department of Biochemistry, Molecular Biology, and Biophysics, University of Minnesota, Minneapolis, Minnesota, United States of America; 2 Department of Biology, Pacific University Oregon, Forest Grove, Oregon, United States of America; 3 Department of Genetics, Cell Biology and Development, University of Minnesota, Minneapolis, Minnesota, United States of America; 4 Department of Molecular Biology and Genetics, Cornell University, Ithaca, New York, United States of America; Indian Institute of Science Education and Research Mohali, INDIA

## Abstract

Toys are us (Trus) is the *Drosophila melanogaster* ortholog of mammalian Programmed Cell Death 2-Like (PDCD2L), a protein that has been implicated in ribosome biogenesis, cell cycle regulation, and oncogenesis. In this study, we examined the function of Trus during *Drosophila* development. CRISPR/Cas9 generated null mutations in *trus* lead to partial embryonic lethality, significant larval developmental delay, and complete pre-pupal lethality. In mutant larvae, we found decreased cell proliferation and growth defects in the brain and imaginal discs. Mapping relevant tissues for Trus function using *trus RNAi* and *trus* mutant rescue experiments revealed that imaginal disc defects are primarily responsible for the developmental delay, while the pre-pupal lethality is likely associated with faulty central nervous system (CNS) development. Examination of the molecular mechanism behind the developmental delay phenotype revealed that *trus* mutations induce the Xrp1-Dilp8 ribosomal stress-response in growth-impaired imaginal discs, and this signaling pathway attenuates production of the hormone ecdysone in the prothoracic gland. Additional Tap-tagging and mass spectrometry of components in Trus complexes isolated from *Drosophila* Kc cells identified Ribosomal protein subunit 2 (RpS2), which is coded by *string of pearls (sop)* in *Drosophila,* and Eukaryotic translation elongation factor 1 alpha 1 (eEF1α1) as interacting factors. We discuss the implication of these findings with respect to the similarity and differences in *trus* genetic null mutant phenotypes compared to the haplo-insufficiency phenotypes produced by heterozygosity for mutants in Minute genes and other genes involved in ribosome biogenesis.

## Authors summary

Ribosomes are essential macromolecular machines required for decoding mRNA to make proteins, the major biomolecules that carry out all central cellular functions. As such, their structural and operational integrity is critical to organismal survival, and mutations that disrupt proper stoichiometry or assembly of ribosomes produce serious pathological consequences during an organism’s development and/or adult life. The ribosome assembly factor PDCD2L is highly conserved from yeast to human, yet its overall function and requirement during development are poorly understood. By examining the developmental consequences of null mutations in *trus*, which encodes the *Drosophila* PDCD2L ortholog, we demonstrate an essential role for this factor in cell-cycle regulation. Furthermore, disruption of Trus function in mitotically dividing imaginal tissue activates the Xrp1-dilp8 stress response pathway which limits production of ecdysone, the major arthropod molting hormone, leading to severe developmental delay during larval stages. These studies provide new insights on the requirements of this highly conserved ribosome assemble factor during development.

## Introduction

Ribosomes are fundamental macromolecular machines present in all life forms that are required for decoding the genome to build cells that have specific identities and functions. As such, their assembly is subject to strict quality control [1] and when aberrations occur, cellular dysfunction and organismal disease are frequent outcomes. In humans, defects in ribosome assembly and subunit production are collectively known as ribosomopathies and produce a myriad of pathologies including microcephaly, intellectual disability, neurodegeneration, seizures, various types of cancers and numerous additional syndromes [2].

Over one hundred years ago, *Drosophila* offered the first insight into the importance of proper ribosome biogenesis, through the isolation of haplo-insufficient *‘Minute’* mutants which, as heterozygotes (*M/+*), develop with a distinctive thin and small bristle phenotype [3]. These heterozygous mutants also exhibit developmental delay, occasional notched eyes, and reduced viability and fertility, while homozygous mutants die at early developmental stages. *M/ + *mutations were subsequently shown to almost exclusively affect ribosomal protein subunits (RPS) [4–6]. Subsequent work uncovered the interesting phenomenon of cell competition whereby slow growing *M/ +* cells (losers), when they are induced as “mosaic clones” in a field of wild-type cells (winners), undergo apoptosis and are eliminated [7]. Cell competition is not just confined to *M/ +* mutations but is observed in similar context-dependent elimination of viable cells in *Drosophila* imaginal epithelia when there exists a discrepancy in genotype between neighboring cells such as heterozygosity for apicobasal polarity genes (*scrib, dlg*), endocytosis components (*Vps25*, *Rab5*), ER stress, and others (reviewed in Nagata and Igaki, 2024) [8]. Cell competition has also been documented in mice, zebrafish, and mammalian tissue culture cells [9–12] suggesting that it may be a universal mechanism for detecting and eliminating growth-compromised clones of cells in an otherwise healthy tissue.

While the molecular mechanism(s) responsible for the various types of cell competition are still not completely understood, in the case of *M/ +* mutants, several recent studies have shown that they activate a novel stress response pathway involving RpS12-mediated induction of the transcription factor Xrp1 [13–16]. Xrp1, likely in complex with another basic leucine-zipper protein (bZIP) Irbp18 [17], then activates downstream targets including JNK-mediate apoptotic genes, DNA damage repair pathways, and antioxidant genes [18]. In addition, Xrp1 stimulates expression of protein kinase R (PKR)-like endoplasmic reticulum kinase (PERK) which then phosphorylates eukaryotic translation initiation factor 2A (EIF2A) leading to a reduction in protein translation in the loser cells and their eventual loss by apoptosis [19–21].

One of the most highly induced Xrp1-dependent genes in *M*/ +cells is *dilp8* [22,23]. This secreted insulin/relaxin-related factor is released from *M/ +* imaginal disc cells and inhibits production of neuronally-derived PTTH, the principal neuropeptide that sets the pace of larval developmental maturation through stimulation of ecdysone production in the prothoracic gland (PG) [14–16]. Knockdown of either *Xrp1* or *dilp8* in *M/ +* cells is sufficient to restore developmental timing to a near normal pace suggesting that Dilp8 activity is the primary mechanism responsible for producing delayed development of *M/ +* larvae.

In addition to the phenotypes caused by mutations in structural subunits of ribosomes, related phenotypes are often produced by mutations in other aspects of ribosome biogenesis. For example, in *Drosophila, RNAi*-mediated knockdown or genetic mutations in components of the nucleolus including Nop60b, Nop140 and Noc1, or Rpl-135, a subunit of the Pol I RNA transcription complex, and Paip1, a poly A binding protein that stimulations translation initiation, can also result in reduced growth and developmental delay [24–27]. In the case of Noc1, *RNAi* mediated knockdown in the wing imaginal disc also resulted in upregulation of *dilp8* via *Xrp1* activation similar to what is seen in *Minute* mutations and likely accounts for the slow development phenotype [28].

Several other well-studied regulators of ribosome biogenesis are vertebrate 40S ribosomal protein uS5/RPS2 and its interaction partners PDCD2 and PDCD2L [29]. In *Drosophila*, the uS5/RPS2 ortholog is encoded by the *string of pearls* (*sop)/RpS2* gene, while the orthologs of PDCD2 and PDCD2L are encoded by *Zfrp8* (*Zinc finger protein RP-8*) and *trus,* respectively [30–32]. The moniker *sop* refers to the oogenesis defect seen in females from recessive hypomorphic sterile alleles which block oocyte development leading to a logjam accumulation of pre-oocytes within each ovariole. These mutants also exhibit classic *Minute-like* phenotypes such as thin bristles and developmental delay [30].

Loss of *uS5/RPS2* in yeast and human cell lines leads to reduction in processing of pre-20S and 21S rRNA precursors, respectively, and defects in nuclear export of pre-40S complexes [33,34]. Biochemical pull-down experiments from yeast and human cell lines identified uS5 as a binding partner of both PDCD2 and PDCD2L [35–38]. These proteins are paralogs that appear to have arisen through gene duplication before the split of animals from plants and fungi [39]. In mice, loss of PDCD2 leads to a failure of the Inner cell mass development after implantation likely due to reduced viability and proliferation of embryonic stem cells [40], while mutants of PDCD2L develop further but die and are resorbed at around day E12.5 [41]. Studies in human cell lines and yeast suggest that PDCD2 and PDCD2L bind to common or overlapping sites on uS5 and act as either a chaperone or an adaptor to facilitate several distinct steps of pre-40S ribosomal particle assembly and transport, and thus are essential for 40S ribosomal subunit biogenesis [29,35,37].

In *Drosophila*, loss of Zfrp8, the PDCD2 homolog, leads to several developmental defects including delayed larval growth, lymph gland over-proliferation, and reduced germ cell proliferation followed by oocyte arrest and degeneration [31,42,43]. Mass spectrometry analysis of complexes containing Tap tagged Zfrp8 identified 30 potential binding partners including Nop60B and uS5/Sop, 5 other ribosomal subunits, several translational elongation and initiation factors and FMRP the fragile-X mental retardation protein [44]. The variety of complexes formed by Zfrp8 suggest that it is likely involved in numerous other molecular processes aside from acting as a chaperone of uS5. Consistent with this view, Zfrp8 is required for proper localization of FMRP in oocytes where the complex likely targets select mRNAs for repression. Interestingly, Zfrp8 and FMRP also appear to regulate heterochromatin formation and transposon de-repression; however, the molecular details for how the various Zfrp8 complexes affect this are still unclear [44].

In this report, we analyze in detail the phenotypes associated with Toys are us (Trus), the *Drosophila* ortholog of mammalian PDCD2L. We compare the various *trus* mutant phenotypes with those produced by *Minute*, *Zfrp8,* and knockdown of other ribosome assembly factors and find both striking similarities but also notable differences, suggesting that Trus loss triggers common ribosomal/proteostasis stress pathways such as Xrp1-Dilp8, but also produces distinctive phenotypes indicative of its specific role in ribosome assembly or its requirement in other biological/developmental processes.

## Results

### Production of *trus* CRISPR/Cas9 mutants

**Fig 1:**
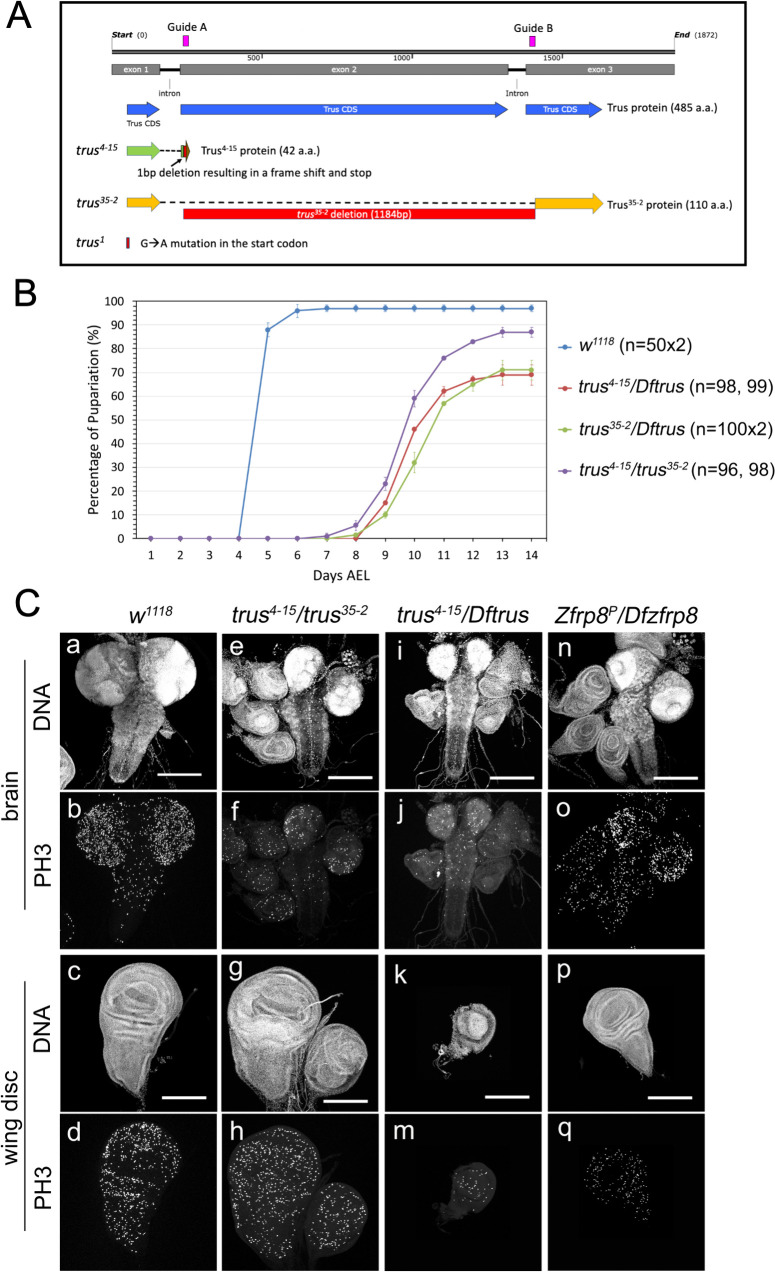
CRISPR/Cas9 induced *trus* mutations cause developmental delay and defects in tissue growth and cell proliferation during the larval stage. (A) A diagram showing *trus* genomic region and *trus* mutant alleles that are used in this study. Trus CDS is shown in blue. Two guide RNAs were designed to flank the entire exon2 and the following intron (magenta). *trus*^*4-15*^ has a single bp deletion at 3R:12,456,811 within target A (red). This results in a truncated 42 amino acid peptide with identity up to Arg40, two missense codons, and a premature stop (green+red+brown). Since this peptide is so small even if it is stably expressed, we consider *trus*^*4-15*^ to be a *trus* null allele. A second allele, *trus*^*35-2*^ is a deletion of 1184 bp between 3R:12,456,805 and 3R:12,457,988 starting 3 bp upstream of target A and ending 5 bp downstream of target B (red). This deletion might give rise to a shorter 110 amino acid peptide lacking residues between Trp38 and Phe414 if stable (orange). Since *trus*^*1*^ allele has a point mutation that alters the initiation codon and the mutant behaves like antimorph, it is possible that the mutation results in an aberrant translational initiation from one of the alternative initiation sites and produces a short fragment. There are two additional candidate initiation sequences which weakly align to the Kozak consensus [90,91] and are in-frame with the Trus protein sequence. Candidate 1 is from +381 bp downstream and candidate 2 is from +579 bp downstream of the original initiation start codon and could produce N-terminally truncated 238 a.a. (~27kDa) or 173 a.a. (~19kDa) Trus fragments, respectively. (B) Pupariation timing of *trus* mutant and *w*^*1118*^. Each data point on the graph indicates an average pupariation percentage of two separate plates. The vertical line on each data point indicates the standard deviation. Numbers of 1st instar larvae picked at 1 Day AEL are shown in parentheses after the genotype. Percentage of pupariation at 14 days AEL were 97%, 87%, 69%, 71% for *w*^*1118*^, *trus*^*4-15*^*/trus*^*35-2*^, *trus*^*4-15*^*/Dftrus*, and *trus*^*35-2*^*/Dftrus*, respectively. After pupariation, most of the *trus* mutant larvae did not develope further and eventually died (pre-pupal lethal) except for some *trus^4-15^/trus^35-2^* that developed to pharates but did not eclose. More than 95% of the *w*^*1118*^ animals eclosed as adults. (C) Representative images of brains and wing discs that were dissected from third instar wandering larvae. Genotypes indicated at the top of the panels. DNA was stained with DAPI and mitotic cells are detected with anti-phospho-HistoneH3 (PH3) antibody. Maximum intensity Z-projections are shown. Scale bar: 200μm.

The original *trus*^*1*^ mutant (*toys are us*) was isolated from the Zucker EMS mutant collection [32,45]. The mutation caused significant developmental delay throughout development and 3rd instar larvae wandered up to 10 days before pupariating and subsequently dying as pre-pupae [32]. Sequencing revealed that the *trus*^*1*^ allele carries a point mutation in the start codon, which may result in a failure of translational initiation (Fig 1A) [32]. We found that a small percentage of heterozygous *trus*^*1*^*/Dftrus* (*Df(3R)BSC847*) developed to pharates and a few enclosed as adults that showed notched eyes and thin/short bristles (S1 Fig). These phenotypes, in addition to the prolonged developmental time, resemble the haplo-insufficiency *‘Minute’* syndrome that is often observed in flies carrying a mutation in one of the genes encoding ribosomal proteins [6]. Since no adult escapers were obtained from *trus*^*1*^ homozygous, we inferred that *trus*^*1*^ might be an antimorph allele. This could arise from an additional unknown mutation on the *trus*^*1*^ chromosome or an aberrant translational start at one of the methionine codons downstream of the normal initiation codon which would produce an N-terminally truncated protein (Fig 1A).

Given the potential complications associated with a phenotypic analysis of the *trus*^*1*^ allele, we generated new *trus* null alleles using CRISPR/Cas9 targeted mutagenesis and isolated two alleles, *trus*^*4-15*^ and *trus*^*35-2*^, that are used in this study (Fig 1A) [46]. Western blot analysis using a polyclonal antibody raised against full-length recombinant Trus showed a band migrating around 60 kDa, corresponding to the full-length Trus protein (predicted Trus molecular weight is 53.2 kDa), that is missing in *trus*^*4-15*^*/trus*^*4-15*^, *trus*^*35-2*^*/trus*^*35-2*^, and *trus*^*1*^*/trus*^*1*^ larval extracts, while the protein level was reduced in heterozygous *trus*^*4-15*^/*TM6*, *trus*^*35-2*^/*TM6*, and *trus*^*1*^/TM6 larval extracts (S2 Fig). We detected no additional truncated fragments that reacted to the anti-Trus antibody on our Western blot (S2 Fig); however, it remains possible that the anti-Trus antibody that was raised against full length of Trus does not react to N-terminally truncated shorter fragments and since *trus*^*35-2*^ and/or *trus*^*1*^ could produce truncated fragments, these may be responsible for their hypomorphic or antimorphic phenotypes, respectively (described below).

### *trus* mutants show developmental delay and pre-pupal lethality

We examined the phenotype of the CRISPR/Cas9 *trus* mutants and first noticed that the mutant animals displayed higher embryonic lethality compared to the control animals (Table 1). We determined the hatch rate of *trus*^*4-15*^*/Dftrus* embryos to be 35% (n = 101), whereas the hatch rate of *Dfd-GMR-nvYPF* positive embryos was 88% (n = 91) (Table 1). Transheterozygous *trus*^*4-15*^*/Dftrus*, *trus*^*35-2*^*/Dftrus*, or *trus*^*4-15*^*/trus*^*35-2*^ mutants that hatched exhibited extensive developmental delay and pupariated at 10–12 days AEL (After Egg Lay), which was 5–7 days later than *w*^*1118*^ (Fig 1B). Some of the larvae remained in the wandering stage for up to 7 days, consistent with the phenotype observed with *trus*^*1*^*/Dftrus* mutants. We noticed that *trus*^*4-15*^*/Dftrus* and *trus*^*35-2*^*/Dftrus* larvae did not actively crawl to a high position on the vial wall during the 3rd instar stage but instead stayed near the food surface. Although *trus*^*4-15*^*/Dftrus*, *trus*^*35-2*^*/Dftrus*, and *trus*^*4-15*^*/ trus*^*35-2*^ produced significant embryonic lethality, those that hatched to become 1st instar larvae largely survived through the larval stages and the pupariation rate of the different mutant allele combinations reached 70–85%, although only after substantial developmental delay. (Fig 1B). After pupariation, most of the larvae died without becoming pupae. Noticeably, the pupariation rate of *trus*^*4-15*^*/ trus*^*35-2*^ animals were 15% higher than *trus*^*4-15*^*/Dftrus* and *trus*^*35-2*^*/Dftrus* (Fig 1B), and some of these pupated and developed to pharate adults, although none eclosed.

**Table 1:** *trus* mutant displays higher embryonic lethality.

Marker	Genotype	plate #	# of embryos aligned	hatched embryos	% hatched
*Dfd-GMR-nvYFP-*	*trus* ^*4–15*^ */Dftrus*	1	41	14	35%
	*trus* ^*4–15*^ */Dftrus*	2	60	21	35%
				Average	35%
*Dfd-GMR-nvYFP+*	*trus* ^*4–15*^*/TM6B P[Dfd-GMR-nvYPF]Sb* *or* *Dftrus/TM6B P[Dfd-GMR-nvYPF]Sb*	3	40	36	90%
	*trus* ^*4–15*^*/TM6B P[Dfd-GMR-nvYPF]Sb* *or* *Dftrus/TM6B P[Dfd-GMR-nvYPF]Sb*	4	51	44	86%
				Average	88%

A cage was set up for a cross between *trus* ^*4-15*^*/TM6B P[Dfd-GMR-nvYPF]Sb* and *Dftrus/TM6B P[Dfd-GMR-nvYPF]Sb*. Embryos were collected on apple-juice/agar plates with yeast paste for 12 hrs. Embryos were sorted based on *Dfd-GMR-nvYFP* plus or minus and separately transferred and aligned along a line of yeast paste on new apple juice/agar plates. The embryo hatch rates were counted 36–48 hrs. AEL.

The *trus*^*4-15*^*/Dftrus* trans-heterozygous combination is a protein null based on genomic sequencing and Western blotting results (S2 Fig), and it produces consistent pre-pupal lethality and developmental delay. We used this genotype as representative of the ‘*trus* zygotic null mutant phenotype’ in most of our subsequent experiments.

### *trus* mutants show defects in tissue growth and cell proliferation

To determine what causes the developmental delay and lethality in *trus* mutants, we first dissected third instar wandering larvae just before pupariation and performed immuno-fluorescent staining of imaginal discs and brains with anti-phospho-HistoneH3 (anti-PH3) antibody and 4’,6-diamidino-2-phenylindole (DAPI). Fig 1C shows representative confocal images of brains (top two rows) and wing discs (bottom two rows) from wild type (Fig 1C, a–d: *w*^*1118*^) and *trus* mutants (Fig 1C, e–m). We first noticed that in *trus*^*4-15*^*/trus*^*35-2*^ and *trus*^*4-15*^*/Dftrus* larvae, brains were significantly smaller than wild-type, and the brain lobes looked unstructured, meaning there was no characteristic ring-structure in the optic lobes as normally appears in wild-type brain lobes (Fig 1C, e and i, compared to a). Their ventral nerve cords (VNC) were narrower and elongated, and the surface of the entire brain appeared disrupted (Fig 1C, e and i).

We also examined tissues from *Zfrp8*^*P*^*/DfZfrp8 (Df(2R)BSC356)* larvae to determine whether the phenotype of *Zfrp8* mutant larvae is similar to that of the *trus* mutant (Fig 1C, n–q). *Zfrp8*^*P*^ is a P-element insertion in the 5’-UTR of the *Zfrp8* gene, and it is not a protein null, therefore it is expected to show a mild phenotype [47]. *Df(2R)BSC356B* is a deficiency chromosome that lacks 42 genes including *Zfrp8*. Brains from *Zfrp8*^*P*^*/DfZfrp8 (Df(2R)BSC356)* larvae (Fig 1C, n) were smaller than wild type (Fig 1C, a) but not as small as *trus* mutants (Fig 1C, e and i). The *Zfrp8* null allele *Df(SM)206* combined with the *Zfrp8*^*M-1-1*^ allele that deletes part of the *Zfrp8* gene (*Df(SM)206/Zfrp8*^*M-1-1*^) is lethal during the early larval stage; therefore we were unable to obtain 3rd instar larval tissue to analyze [31].

**Fig 2:**
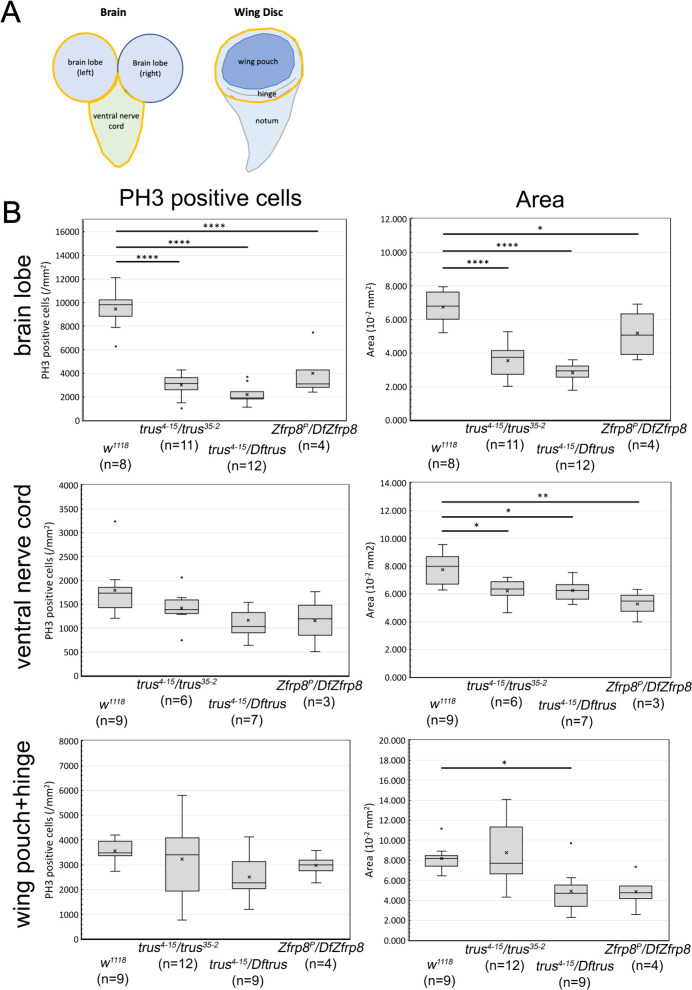
*trus* mutation caused a significant reduction of mitotic cell number and size of brain and wing disc. (A) Diagrams showing the larval brain and wing-disc. The areas surrounded by yellow lines indicates brain lobe, ventral nerve cord, and wing pouch plus hinge areas that are quantified in B. (B) Quantification of PH3 positive cells/mm^2^ (left column) and area in mm^2^ (right column) for brain lobe, ventral nerve cord, and [wing pouch + hinge] are shown. Genotypes and number of tissues measured for each genotype are indicated in parenthesis under the graphs. The x in the boxes indicates the mean value and the line inside the box indicates the median. Box-and-whisker plots were generated using Microsoft Excel (Redmond, WA) and ANOVA analysis followed by Dunnett’s test to compare each mutant to wild-type (*w*^*1118*^) were performed using Prism 10 (GraphPad Software, INC., Boston MA). When the P-value in Dunnett’s test indicates that the pair variance is statistically significant, it is shown as a horizontal line across the *w*^*1118*^ and the mutant with * (P<=0.05), ** (P<=0.01), *** (P<=0.001), or **** (P<=0.0001) (data in S2 File). PH3 positive cells and area measurements of [wing pouch+hinge] from *trus*^*4-15*^*/trus*^*35-2*^ larvae show significant variations within the group, some wing discs are small, and others are overgrown, suggesting that the *trus*^*35-2*^ allele that possibly produces a 110 a.a. Trus fragment is causing hypo/hyper-morphic effects in combination with the protein null *trus*^*4-15*^ allele. The observed variation is not likely to be caused by experimental errors but is merely indicative of the *trus*^*4-15*^*/trus*^*35-2*^ mutant phenotype. When we removed the intrinsically variable *trus*^*4-15*^*/trus*^*35-2*^ group from ANOVA analysis of [wing pouch+hinge], the results for PH3 positive cell count and area measurement showed P < 0.05 (*) and P < 0.01 (**), respectively. Following Dunnett’s tests indicated P < 0.01 (**) for both PH3 count and area comparisons between *trus*^*4-15*^*/Dftrus* vs. *w*^*1118*^ (see ANOVA analysis 2 in S2 File). This indicates that wing disc growth is clearly inhibited in *trus* null mutant (*trus*^*4-15*^*/Dftrus*) compared to *w*^*1118*^. The Dunnett’s test also showed that area of [wing pouch+hinge] from *Zfrp8*^*P*^*/DfZfrp8* larvae compared to *w*^*1118*^ was significantly smaller with P < 0.05 (*).

We next examined the imaginal discs including leg, haltere, and wing discs from *trus*^*4-15*^*/Dftrus* larvae (Fig 1C, i and k) and found that they were smaller than wild type (Fig 1C, c), with wing discs showing the most severe growth impairment and morphological defects. The discs were under-developed and too small to dissect out from early wandering larvae; however, after additional development during the prolonged wandering stage, the wing discs reached a size that could be dissected and further examined. Intriguingly, wing discs from *trus*^*4-15*^*/trus*^*35-2*^ larvae developed inconsistently during the late wandering stage. Some were larger than wild type and often showed excessive folds, whereas the others were smaller than wild type, so the overall size distribution was diverse. Wing discs from *Zfrp8*^*P*^*/DfZfrp8* were less affected than *trus*^*4-15*^*/Dftrus* but were still smaller than wild type (Fig 1C, c and p). Fig 2B shows quantification of PH3 positive cells (number/mm^2^) and area (mm^2^) of brain lobe, ventral nerve cord, and wing pouch plus hinge as indicated with yellow-line enclosed areas in Fig 2A. In brain lobes from *trus*^*4-15*^*/Dftrus* and *trus*^*4-15*^*/trus*^*35-2*^ larvae, PH3 positive (mitotic) cell numbers were significantly less than *w*^*1118*^ control, and the area (size) of brain lobes was also significantly smaller than *w*^*1118*^. We observed less significant changes of PH3 count of ventral nerve cords from *trus*^*4-15*^*/Dftrus* and *trus*^*4-15*^*/trus*^*35-2*^, however, area reductions were still significant for the *trus* mutants comparing to wild type. We saw significant decrease of PH3 positive cells in brain lobes from *Zfrp8*^*P*^*/DfZfrp8.* The quantification confirmed a significant reduction of area of wing pouch + hinge from *trus*^*4-15*^*/Dftrus* compared to *w*^*1118*^ (Fig 2B, and additional information in the accompanying figure legend).

In contrast to the absolute pre-pupal lethality of *trus*^*4-15*^*/Dftrus* larvae, we observed rare escapers from *trus*^*4-15*^*/trus*^*35-2*^ larvae that developed to pharate adults but did not eclose. The size and cell proliferation variances observed in wing discs from *trus*^*4-15*^*/trus*^*35-2*^ animals are consistent with the variable penetrance in the pre-pupal/pharate lethality of the mutant. We speculate that the *trus*^*35-2*^ allele may produce a truncated Trus protein product that has partial function and appears to trigger metamorphosis of a limited number of mutant larvae allowing them to progress into the pupal-pharate stage.

Given the observed structural abnormalities and extensive reduction of brain and wing disc size in *trus* mutants (Fig 1C), we investigated whether apoptosis contributes to the tissue growth defects in the mutants and whether this ultimately leads to developmental delay and lethality. As we could not detect an increase in apoptosis in *trus* mutant brain and wing discs by anti-cleaved caspase3 antibody staining or TUNEL assay, we further examined whether inhibiting apoptosis by ubiquitously overexpressing baculovirus p35, a caspase inhibitor [48,49], with *daughterless-GAL4* could rescue the defects in tissue growth, cell proliferation, and developmental timing of *trus* mutants, and found that it could not (S4 Fig). Our results suggest that loss of Trus does not trigger widespread apoptosis, rather the developmental and tissue growth abnormalities likely reflect a deficiency in the control of cellular proliferation.

### *RNAi* mediated *trus* knockdown in wing discs causes growth and cell proliferation defects

**Fig 3:**
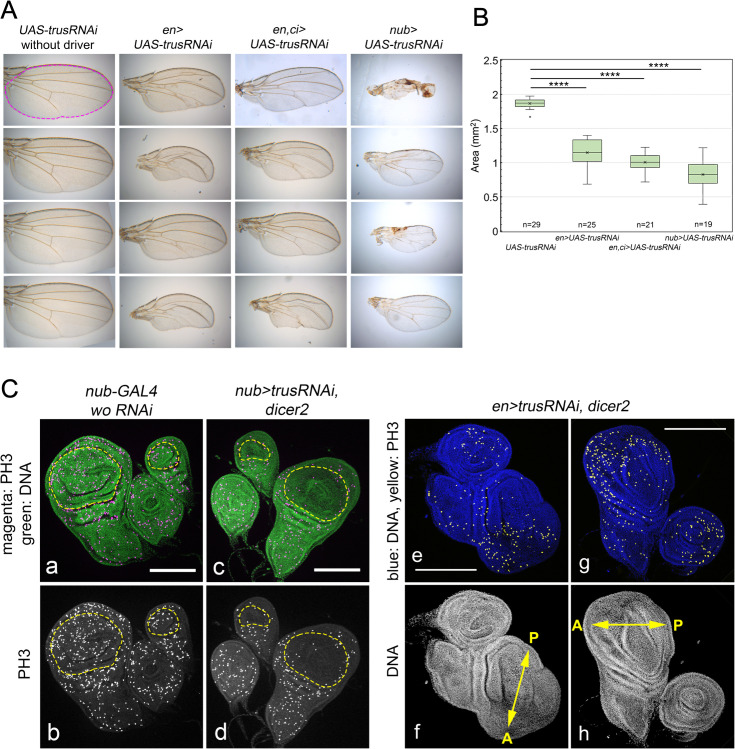
*trusRNAi* induced with various wing disc drivers affects wing size and morphology and decreases cellular proliferation. (A) Representative images of wings from female adult flies that had *Trus RNAi* induced with wing disc drivers. Adult wings from flies carrying *UAS-trusRNAi* without any driver, *en-GAL4* driven *UAS-trusRNAi*, *en* and *ci-GAL4* driven *UAS-trusRNAi*, or *nub-GAL4* driven *UAS-trusRNAi* are shown. (B) Quantification of wing area calculated in mm^2^. An example area is represented as magenta dashed outline in the top-left in A. ImageJ/Fiji (https://imagej.net) was used for area measurement. Box-and-whisker plots were generated using Microsoft Excel. The x in the boxes indicates the mean value and the line inside the box indicates the median. Sample numbers are indicated at the bottom of the graph (ex. n = 29 for *UAS-trusRNAi*). ANOVA analysis followed by Dunnett’s test to compare wing area of each RNAi samples to *w*^*1118*^ were performed using Prism 10 (GraphPad Software, INC.). **** indicates P value <=0.0001 (S3 File). (C) Reduction of anti-PH3 stained foci is observed in the pouch area of wing and haltere discs in *nub>trusRNAi, UAS-dicer2* larvae (a-d) and the posterior half of wing discs and one half of the leg disc in *en>trusRNAi, UAS-dicer2* larvae (e-h). In a and c, green: DAPI staining and magenta: anti-PH3 staining. Yellow dashed lines indicate pouch of wing and haltere discs. In e and g, blue: DAPI staining and yellow: anti-PH3 staining. Arrows in f and h indicate the anterior (A)- posterior (P) axis of wing discs. For each genotype, more than 12 wing discs were dissected and they showed the similar phenotypes. Maximum intensity Z-projections are shown. Bar: 200μm.

To help distinguish autonomous verses non-autonomous effects of *trus* loss on imaginal disc growth, we used RNAi knockdown mediated by different imaginal disc Gal4 driver lines including *engrailed-GAL4* (*en-GAL4*), *en-GAL4* and *cubitus-interruptus-GAL4* (*ci-GAL4*) simultaneously, or *nubbin-GAL4* (*nub-GAL4*). Fig 3A shows resulting wing phenotypes, in which *nub-GAL4* caused an overall wing size reduction (*nub > UAS-trusRNAi*), and *en-GAL4* (*en > UAS-trusRNAi*) or *en-GAL4* and *ci-GAL4* (*en,ci > UAS-trusRNAi*) caused disruption of wing veins and reduction of wing area, especially in the posterior part of wings compared to the control (*UAS-trusRNAi* without a driver) (Fig 3A). Quantification of the wing area using ImageJ indicates that the reduction in wing size is significant with either wing driver (Fig 3B). Consistent with the reduced size, we found that *trusRNAi* caused a reduction of proliferation in larval wing discs specific to the area where *trusRNAi* was expressed (Fig 3C). The expression pattern of each *GAL4* driver used in the *trusRNAi* experiments and later used in *trus* mutant rescue experiments can be found in S3 Fig.

To analyze expression patterns in detail, we chose a fast-maturing nuclear reporter *UAS-RedStinger* (*DsRed.T4.NLS*) [50]. We confirmed that *nub-GAL4* is only expressed in the pouch area of wing and haltere discs, *en-GAL4* is expressed only in the posterior half of wing and haltere discs, and *ci-GAL4* is expressed in the anterior half of wing and haltere discs, as reported previously [51–53] (S3 Fig, c, d, k, m, p, and q). We observed that *nub-GAL4* driven *trusRNAi* leads to a reduction of mitotic cell number detected by anti-PH3 antibody only in the pouch of wing and haltere discs, resulting in a reduced pouch size (Fig 3C, a–d). Knockdown of *trus* using *en-GAL4* reduced the mitotic cell number only in the posterior half of the wing disc and the leg disc, resulting in considerable reduction of the posterior half of the wing disc (Fig 3C, e–h). Our observations indicate that *trus* knockdown inhibits cellular proliferation in a tissue and cell-autonomous manner. We note that *trusRNAi* driven by a combination of *en-GAL4* and *ci-GAL4* affects the posterior part of the wing more than the anterior (*en,ci > UAS-trusRNAi*) (Fig 3A). This can be explained by the fact that when both *en-GAL4* and *ci-GAL4* are used simultaneously, *en-GAL4* expression is much stronger than *ci-GAL4* for unknown reasons (S3 Fig, r-u).

### Loss of *trus* in wing discs causes developmental delay and lethality

**Fig 4:**
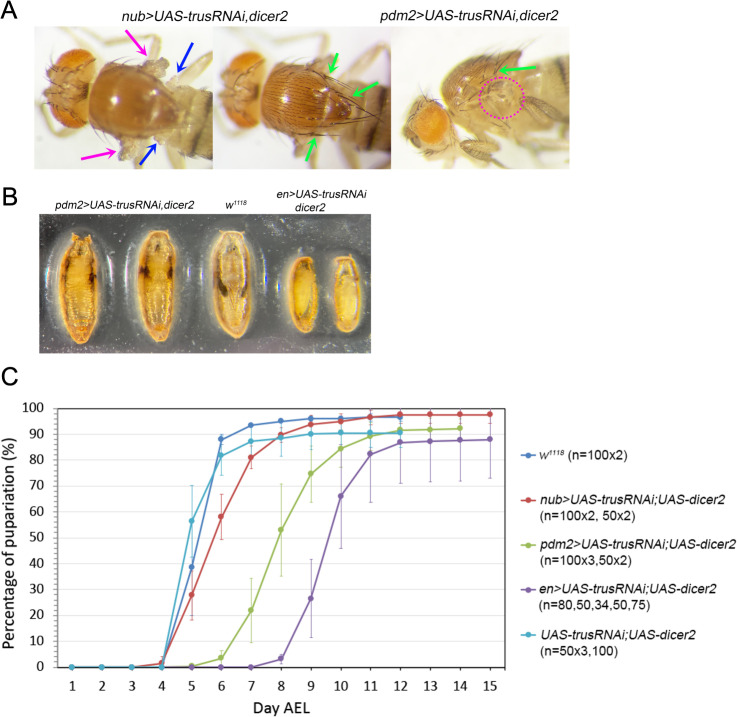
*trusRNAi* induced with wing disc drivers delay pupariation timing. (A) *trusRNAi* induced with either *nub-GAL4* or *pdm2-GAL4* in the presence of *UAS-dicer2* show a complete loss of wing blade (magenta arrows and magenta dotted-circle), morphological defects in halteres (blue arrows), and extra/disorganized bristles (green arrows). (B) (left) *pdm2 > trusRNAi* pharates with wing defects. (right) *en>trusRNAi* larvae pupariate precociously resulting in smaller pre-pupae that never become pupae. (middle) *w*^*1118*^ (control) pupa shown for size and structural comparison. (C) Pupariation timing of *trusRNAi* larvae induced with *nub-GAL4* (red), *pdm2-GAL4* (green), or *en-GAL4* (purple). Pupariation timing of *w*^*1118*^ larvae (blue) and larvae carrying *UAS-trusRNAi* and *UAS-dicer2* without driver (light blue) are shown as controls. Each data point represents an average pupariation percentage from multiple plates. The vertical line on each data point indicates the standard deviation. Numbers of 1st instar larvae picked at 1 Day AEL are shown in parentheses after the genotype.

When we enhanced *trusRNAi* knockdown using *UAS-dicer2*, together with *nub>trusRNAi* or *pdm2 > trusRNAi*, both of which express primarily in the wing pouch, (S3 Fig, c-d, g-h), we noted a complete loss of wing blades leaving a small hinge in adult flies, haltere defects, and extra bristles (Fig 4A). Interestingly, *dicer2*-enhanced *nub>trusRNAi*, *pdm2 > trusRNAi*, and *en>trusRNAi* also resulted in partial lethality during the pre-pupal or pupal stages. The pupariation rate was high with all the drivers; however, the adult eclosion rate of *pdm2 > trusRNAi* decreased to ~10% and *nub>trusRNAi* to ~30%, while the eclosion rate without a driver averaged 79% (Table 2). Notably, *en>trusRNAi* resulted in a high rate of embryonic lethality, and the surviving larvae were 100% pre-pupal lethal (Table 2). In addition, many larvae showed a “Tubby-like” phenotype resulting in significantly smaller pre-pupae than *w*^*1118*^ or *pdm2 > trusRNAi* pupae (Fig 4B). This observation is in line with reports that *engrailed* mutant embryos are Tubby-like and lethal during the embryonic stage [53]. We suspect that *trusRNAi* driven with *en-GAL4* causes inhibition of cell proliferation, specifically in posterior compartments, leading to a failure of posterior segment establishment during embryogenesis similar to *engrailed* mutants, and this likely contributes to the high rate of embryonic lethality.

**Table 2:** Percentage of pupariation and eclosion of larvae that were induced *trus RNAi* in wing discs.

Genotype (number of 1st instar larvae)	number of pupae or pre-pupae (% of larvae)	number of adults eclosed (% of larvae)
*w1118*-1 (n = 100)	95 (95%)	95 (95%)
*w1118*-2 (100)	98 (98%)	94 (94%)
AVERAGE%	97%	95%
*nub > UAS-trusRNAi,UAS-dicer2*–1 (100)	100 (100%)	29 (29%)
*nub > UAS-trusRNAi,UAS-dicer2*–2 (100)	100 (100%)	33 (33%)
*nub > UAS-trusRNAi,UAS-dicer2*–3 (50)	49 (96%)	10 (20%)
*nub > UAS-trusRNAi,UAS-dicer2*–4 (50)	47 (94%)	19 (38%)
AVERAGE%	98%	30%
*pdm2 > UAS-trusRNAi,UAS-dicer2*–1 (100)	97 (97%)	10 (10%)
*pdm2 > UAS-trusRNAi,UAS-dicer2*–2 (100)	86 (86%)	11 (11%)
*pdm2 > UAS-trusRNAi,UAS-dicer2*–3 (100)	92 (92%)	13 (13%)
*pdm2 > UAS-trusRNAi,UAS-dicer2*–4 (50)	47 (94%)	4 (8%)
*pdm2 > UAS-trusRNAi,UAS-dicer2*–5 (50)	46 (92%)	4 (8%)
AVERAGE%	92%	10%
*en > UAS-trusRNAi,UAS-dicer2*–1 (80)	69 (86%)	0%
*en > UAS-trusRNAi,UAS-dicer2*–2 (50)	30 (60%)	0%
*en > UAS-trusRNAi,UAS-dicer2*–3 (34)	34 (100%)	0%
*en > UAS-trusRNAi,UAS-dicer2*–4 (50)	47 (94%)	0%
*en > UAS-trusRNAi,UAS-dicer2*–5 (75)	74 (99.7%)	0%
AVERAGE%	88%	0%
*UAS-trusRNAi;UAS-dicer2*–1 (50)	50 (100%)	42 (84%)
*UAS-trusRNAi;UAS-dicer2*–2 (50)	44 (88%)	44 (88%)
*UAS-trusRNAi;UAS-dicer2*–3 (50)	43 (86%)	34 (68%)
*UAS-trusRNAi;UAS-dicer2*–4 (100)	88 (88%)	74 (74%)
AVERAGE%	91%	79%

Number of first instar larvae indicated in the parentheses after genotype

(ex. n = 100) were selected and transferred to a new regular cornmeal agar fly food plate. Number and percentage (in parentheses) of the larvae that became pre-pupae, pupae, and eclosed adults were counted and indicated in the second and the third columns.

In addition to these morphological defects, we found that *dicer2*-enhanced *trusRNAi* also caused significant larval developmental delay. Without a driver (*UAS-trusRNAi*), pupariation occurred at 5–6 days AEL similar to the *w*^*1118*^ control (Fig 4C light blue and blue). In contrast, *nub>Trus RNAi* larvae pupariated at 5–8 days AEL (delayed 1–2 days compared to the control, Fig 4C red), *pdm2 > trusRNAi* larvae pupariated at 7–10 days AEL (delayed 2–5 days, Fig 4C green), and *en>trusRNAi* pupariated at 9–11 days AEL (delayed 4–6 days, Fig 4C purple).

Since the disc-specific RNAi knockdown of *trus* results largely recapitulates the cell proliferation and developmental delay phenotypes exhibited by genetic null mutants, we infer that it may be the reduced imaginal disc cell proliferation that is key to understanding the developmental delay.

### The Xrp1-Dilp8 stress response pathway is largely responsible for the developmental delay of *trus* mutants

**Fig 5:**
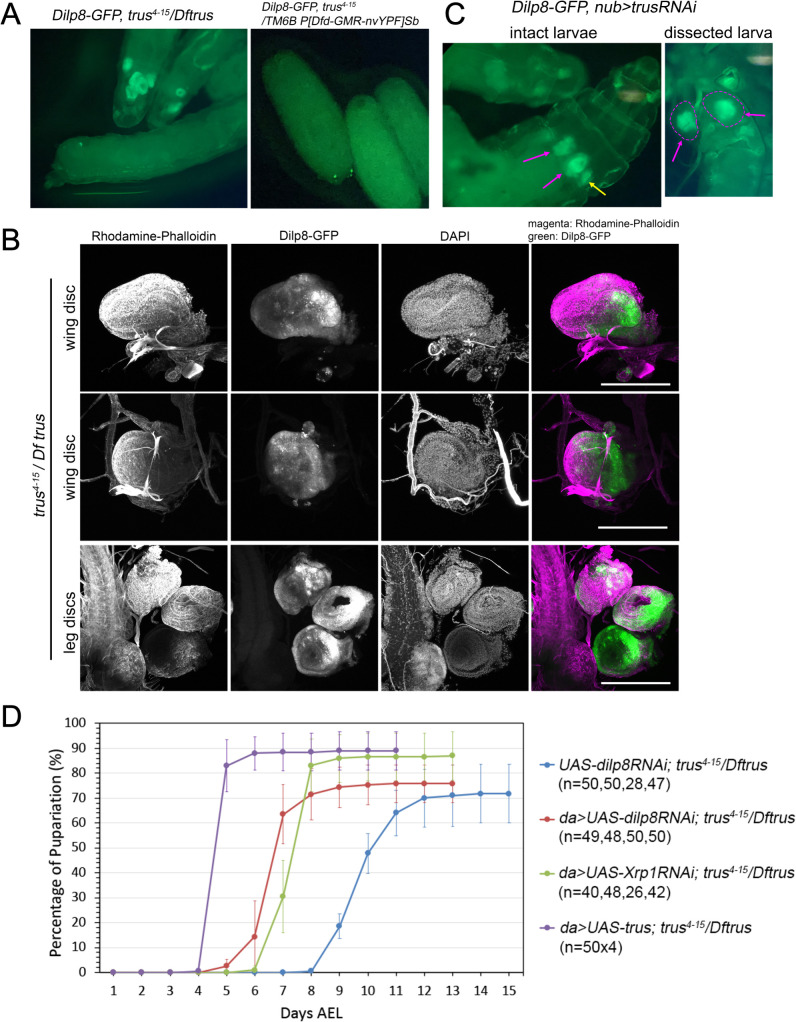
Xrp1-Dilp8 pathway is activated leading to developmental delay in *trus* mutants. (A) (left) Dilp8-GFP expression in third instar wandering *trus* ^*4-15*^*/ Dftrus* larvae. (right) Dilp8-GFP expression in 3rd instar wandering *trus*^*4-15*^*/ TM6B P[Dfd-GMR-nvYPF]Sb or Dftrus/ TM6B P[Dfd-GMR-nvYPF]Sb larvae.* Two bright GFP dots are Dfd-GMR-nvYFP signals on eyes. (B) Fixed and dissected wing (first and second rows) and leg (bottom row) discs from *trus* ^*4-15*^*/ Dftrus* third instar wandering larvae show Dilp8-GFP expression (green). Rhodamine-Phalloidin and DAPI staining reveal significant reduction in size and abnormal morphologies of the discs. 16 larvae were dissected, and representative images are shown. Maximum intensity Z-projections are shown. Scale bar: 200μm. (C) (left) Expression of Dilp8-GFP in the pouch region of the wing disc (magenta arrows) and haltere disc (yellow arrow) from *nub> trusRNAi* larvae. (right) A dissected *nub>trus RNAi* larval wing discs are marked with a magenta dashed line and show GFP fluorescence in the middle area of the wing discs (wing pouch). (D) Developmental timing curves show that *da>dilp8RNAi* (red) or *da > Xrp1RNAi* (green) significantly rescue the developmental delay of *trus*^*4-15*^*/Dftrus* larvae. *da > UAS-trus* rescues the developmental delay and lethality of *trus*^*4-15*^*/Dftrus* to the wild-type level and is shown as the positive control (purple). *trus*^*4-15*^*/Dftrus* larvae with the *UAS-dilp8RNAi* transgene without driver serve as the negative control (blue).

The Xrp1-Dilp8 signaling pathway has been shown to coordinate *Drosophila* tissue growth with developmental timing [15,54,55]. Xrp1 is a stress response transcription factor and is activated in growth impaired tissues promoting production and release of the peptide hormone Dilp8. Dilp8 then acts remotely on Lgr3 positive neurons in the central brain to inhibit the release of PTTH thereby blocking the production of molting hormone ecdysone [56]. Since *trus* mutants show growth and cell proliferation defects in imaginal discs, and are developmentally delayed, we hypothesized that the Xrp1-Dilp8 pathway is the link between tissue growth impairment and slow development in *trus* mutants. To test this hypothesis, we recombined a genomic *GFP* insertion of *dilp8* (*dilp8-GFP*) [57] onto the *trus*^*4-15*^ chromosome. We found that Dilp8-GFP protein is expressed in wing, leg, and genital discs in *trus*^*4-15*^*/Dftrus* larvae, while Dilp8-GFP could not be detected in the control larvae (Fig 5A). As described previously, wing discs and, to some extent, leg discs dissected from these late wandering larvae are small and disorganized but show strong patches of Dilp8-GFP (Fig 5B Dilp8-GFP). We also induced *trusRNAi* with *nub-GAL4* and found that Dilp8-GFP was expressed in the pouch area of wing and haltere discs (magenta and yellow allows in Fig 5C), suggesting that *dilp8* expression is involved in the developmental delay observed with *trusRNAi* described above (Fig 4C).

To examine directly whether the Xrp1-Dilp8 signaling pathway is involved in the developmental delay of *trus* mutants, we individually knocked down *Xrp1* and *dilp8* in *trus*^*4-15*^*/Dftrus* mutants by RNAi induced with *daughterless-GAL4*, a ubiquitous driver. We found that *Xrp1RNAi* and *dilp8RNAi* accelerated the *trus* mutant’s pupariation timing by 4 and 5 days, respectively, compared to the control (Fig 5D). With both *Xrp1RNAi* and *dilp8RNAi*, however, the majority of animals were pre-pupal lethal the same as *trus*^*4-15*^*/Dftrus* mutants without RNAi.

We next asked if in addition to rescue of developmental delay, whether knockdown of *Xrp1* or *dilp8* could also rescue the CNS and imaginal disc cell proliferation defects. We noted that when the *Xrp1RNAi* rescue crosses were kept on vial food, some of the *da > Xrp1RNAi; trus*^*4-15*^*/Dftrus* pre-pupae wandered out of the food and developed to pupae, and a few became pharate adults that did not eclose. This indicates some rescue of the metamorphosis defect since normally *trus* null mutants are 100% pre-pupal lethal and remain close to the food. When we examined the brain and imaginal discs from da > *Xrp1RNAi; trus*^*4-15*^*/Dftrus* third instar wandering larvae with anti-phospho-Histone H3 and DAPI staining, surprisingly, in 10 dissected larvae, cell proliferation in all the brains was significantly rescued compared to the original *trus*^*4-15*^*/Dftrus* brains (S5A Fig), although wing disc defects were only moderately rescued (S5B Fig). We also looked at brain and discs of *Dilp8RNAi* induced *trus*^*4-15*^*/Dftrus* third instar wandering larvae. Again, cell proliferation in the brain lobes appeared to be moderately rescued comparing to original *trus*^*4-15*^*/Dftrus* mutant (S6A Fig) but not as much as the *Xrp1RNAi* induced larvae (S5A Fig). In addition, wing discs defects were not rescued at all by *dilp8RNAi* (S6B Fig). These results suggest that the both the tissue growth/cell proliferation defect and the developmental delay are caused to a large extent by the activation of the Xrp1-Dilp8 stress response pathway.

### Developmental delay of *trus* mutants is rescued by ecdysone feeding

**Fig 6:**
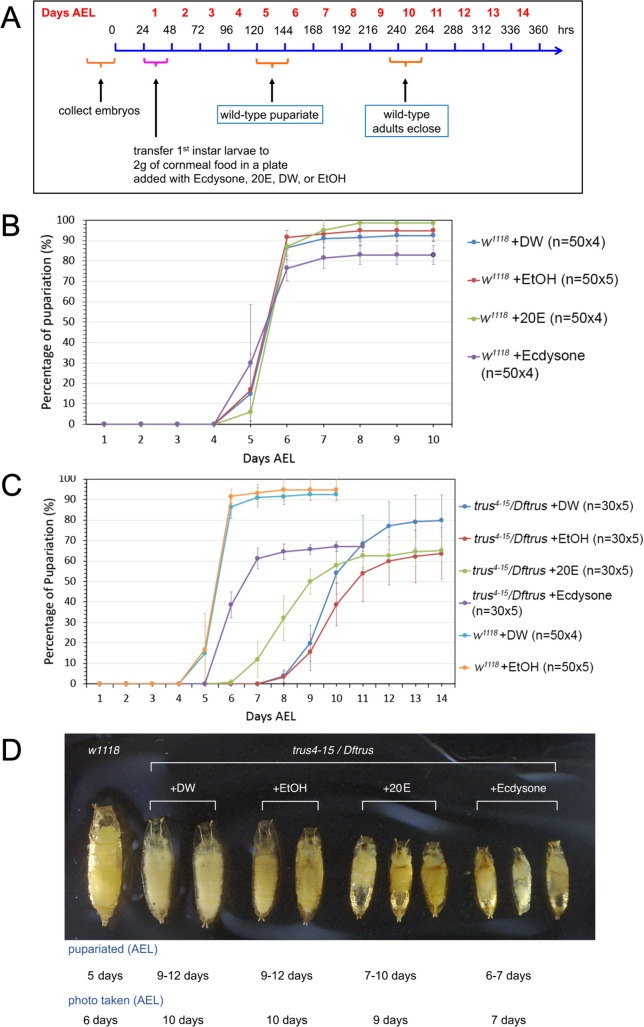
Ecdysone feeding to *trus* mutant larvae accelerates pupariation timing but causes precocious pupariation and does not rescue the pre-pupal lethality. (A) Schedule of the ecdysone-feeding experiment. The larvae were fed mashed regular cornmeal fly food that was mixed with 20E or ecdysone dissolved in solvent (ethanol) beginning at the 1st instar stage (1 day AEL). For the controls, fly food was mixed with either water or ethanol only. (B) Pupariation timing of *w*^*1118*^ larvae fed cornmeal fly food mixed with either ecdysone (purple), 20-hydroxyecdysone (20E) (green), dH_2_O (blue), or ethanol (red). (C) Pupariation timing of *trus*^*4-15*^*/Dftrus* larvae fed cornmeal food mixed with either ecdysone (purple), 20E (green), dH_2_O (blue), or ethanol (red). Pupariation timing of *w*^*1118*^ fed cornmeal food mixed with dH_2_O (light blue), or ethanol (orange) are from the same data set shown in B. Each circle represents the average percentage of pupariated larvae from multiple dishes of the same genotype. Pupariated larvae were counted every 24 hours. Standard deviations are shown as vertical lines for each data point in B and C. (D) Pre-pupae that were fed cornmeal food mixed with either dH_2_O, ethanol, 20E, or ecdysone with the time of pupariation indicated below. *w*^*1118*^ pupa is shown on the left for size comparison.

To demonstrate directly that Xrp1-Dilp8 stress response signal induced in *trus* mutants is causing developmental delay by inhibiting ecdysone production, we fed *trus* mutant larvae with 20-hydroxyecdysone (20E) or its precursor ecdysone. The feeding scheme is outlined in Fig 6A. We found that feeding 20E to *w*^*1118*^ larvae did not affect the pupariation timing (green in Fig 6B) compared to the controls fed with water (blue) or ethanol (red), while feeding the precursor ecdysone slightly accelerated pupariation and reduced overall pupariation rate by 10% (purple) compared to the control or 20E-fed larvae (Fig 6B). This data is consistent with the results reported previously by Ono (2014) where it was shown that feeding ecdysone to wild type larvae after L3 ecdysis accelerated pupariation by 6–12 hours and increased larval lethality [58].

When we examined *trus*^*4-15*^*/Dftrus* larvae fed with regular cornmeal food supplied with water (blue) or ethanol (red), they pupariated at 9–12 days AEL, while *w*^*1118*^ larvae pupariated at 5–6 days AEL. 20E-fed larvae pupariated at 7–11 days AEL, and ecdysone-fed larvae pupariated at 6–7 days AEL (purple) (Fig 6C). Interestingly, feeding ecdysone was much more effective in rescuing the developmental delay of *trus* mutants than feeding 20E, and as suggested by Ono (2014), this may indicate that ecdysone has an unknown function in regulating developmental timing separately from 20E. Despite rescue of the developmental delay, 100% of the *trus*^*4-15*^*/Dftrus* animals that were fed 20E or ecdysone died as pre-pupae without developing into pupae. Notably, however those 20E-fed or ecdysone-fed pre-pupae were significantly smaller than the control mutant larvae (Fig 6D) likely due to accelerated development, as has been noted previously in ecdysone feeding experiments [58]. We conclude that the developmental delay in *trus* mutant larvae is caused by ecdysone deficiency as a result of induction of the Xrp1-Dilp8 stress response pathway.

### The core structure of Trus protein and its orthologs are conserved

**Fig 7:**
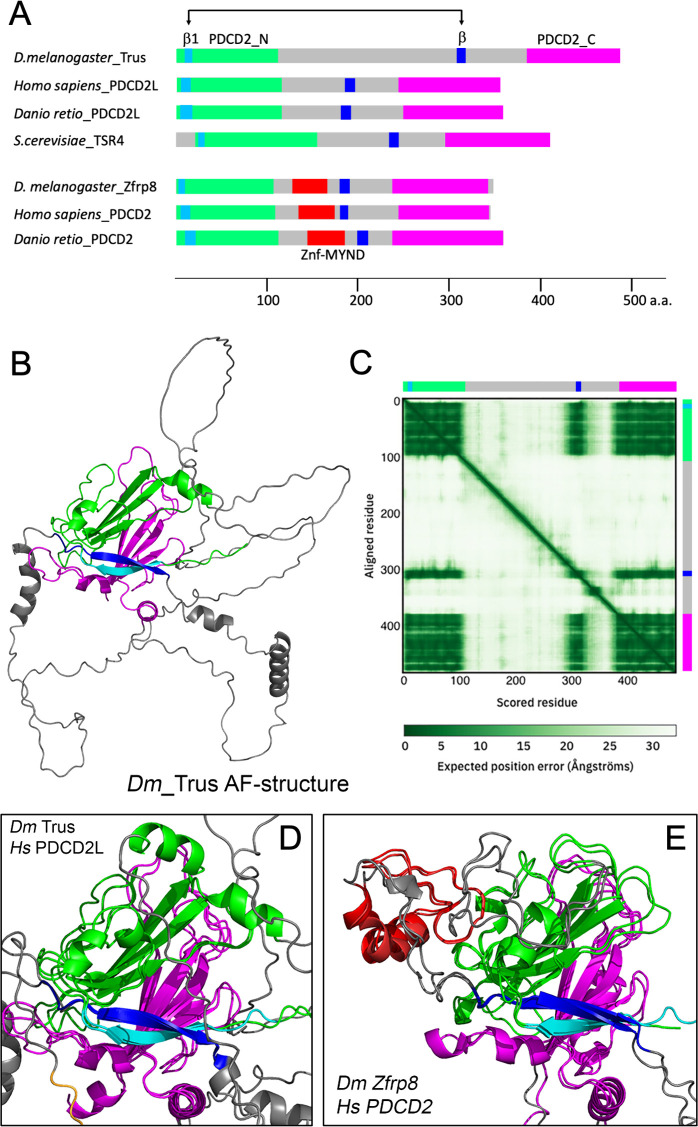
Predicted 3D Structure of Trus and its paralog Zfrp8 share a core module that is conserved through evolution. (A) Domain structure comparison of *Drosophila* Trus, its paralog Zfrp8, and their orthologs from different organisms. *D. melanogaster* Trus (Accession number: Q9VG62; *Dmel*\CG5333), Homo sapiens_PDCD2L (Q9BRP1), *Danio retio*_PDCD2L (Q5RGB3), *S. cerevisiae*_Tsr4 (P87156), *D. melanogaster*_Zfrp8 (Q9W1A3), *Homo sapiens*_PDCD2 (Q16342), and *Danio retio*_PDCD2 (Q1MTH6) are shown. Green: PDCD2_N, magenta: PDCD2_C, red: MYND-type zinc finger domain (Znf-MYND), light blue: first β-strand in PDCD2_N domain, blue: β-strand that is predicted to interact with the first β-strand, gray: unstructured loop. (B) *D. melanogaster* Trus protein 3D structure predicted by AlphaFold (https://alphafold.ebi.ac.uk/). Color-coding of domains are the same as shown in A. The PDCD2_N consists of four β-strands from which three β-strands form a β-sheet structure immediately followed by an α-helix (shown in green). In addition, the first β-strand in the PDCD2_N domain (residues 9-16, shown in light blue) is predicted to form hydrogen bonds with another β-strand (residues 307-317, shown in blue) in the middle of the loop region between the domain PDCD2_N and the domain PDCD2_C. (C) Predicted Aligned Error (PAE) of *Drosophila* Trus 3-D structure calculated by the AlphaFold. Color-coded bars representing the Trus protein domains shown in B are placed on upper and right sides of the panel. The PAE indicates high confidence in the relative position of scored residues 1-109 (PDCD2_N; shown in light blue and green) when aligned with residues 382-485 (PDCD2_C; shown in magenta), supporting the packing between these regions that form a structural module despite the large unstructured loops (gray) that separate the PDCD2_N and PDCD2_C domains. The PAE of the blue β−strands (residues 307-317) against both the N-terminal domain (PDCD2_N, residues 1-109) and the C-terminal domain (PDCD2_C, residues 382-485) shows high confidence, indicating packing of a core module that includes the PDCD2_N (light blue and green, residues 1-109), the PDCD2_C (magenta, residue 382-485), and a β-strand in-between (blue, residues 307-317). (D) Structural alignment of *Dm* Trus and *Hs* PDCD2L shows evolutional conservation of the core module in the PDCD2L protein family. (E) Structural alignment of *Dm* Zfrp9 and *Hs* PDCD2 shows evolutional conservation of the core module in the PDCD2 protein family. 3-D structural alignments were performed with PyMOL (Schrödinger LLC., NY).

Fig 7A shows the domain structures of *Drosophila* Trus and Zfrp8 together with several vertebrate and yeast orthologs. Trus and Zfrp8 have highly conserved N-terminal (shown in green and light blue) and C-terminal (shown in magenta) globular domains. The C-terminal conserved domain was previously relegated to the PDCD2L/PDCD2 protein superfamily and named PDCD2_C in the Pfam protein database [59]. We call the N-terminal conserved domain PDCD2_N in this paper. AlphaFold prediction of the *Drosophila* Trus 3D structure shown in Fig 7B [60,61] reveals a core module that includes two β-sheets facing each other, one consists of three β-strands from PDCD2_N domain (green) and the other consists of four β-strands from PDCD2_C domain (magenta), and a pair of interacting β-strands (light blue and blue) (Fig 7B and 7C).

Notably, structural alignments of *Drosophila* Trus against human PDCD2L (Fig 7D), zebrafish PDCD2L (S7C Fig), and *Saccharomyces cerevisiae* TSR4 (S7D Fig) show striking conservations of the core module through evolution. Structural alignment of *Drosophila* Zfrp8 against human PDCD2 indicates that the core structural module is also conserved through evolution in the Zfrp8/PDCD2 orthologs (Fig 7E). We also note that AlphaFold predictions demonstrate that the core structural module is well aligned between Trus/PDCD2L and Zfrp8/PDCD2 paralogs (S7B Fig), with the latter lacking the α-helix at the end of the PDCD2_N domain having instead a MYND-type zinc finger domain in the loop region outside of the core module that was previously shown to be involved in protein-protein interactions [62] (shown in red in Figs 7E, and S7A and S7B). The core module of the PDCD2L/PDCD2 protein superfamily has been previously annotated as a TYPP domain by Burroughs and Aravind (2014) using extensive comparative genome sequence and structure analytical techniques [39]. The conservation of the core module among TSR4, PDCD2L and PDCD2 orthologs suggests that these proteins exhibit similar biochemical properties, whereas the structural divergence between PDCD2L and PDCD2 paralogs suggests that PDCD2L and PDCD2 may control specific functions or steps within the highly complex process of ribosomal biogenesis (Figs 7 and S7).

### Trus is expressed at high levels in larval mitotic tissues

**Fig 8:**
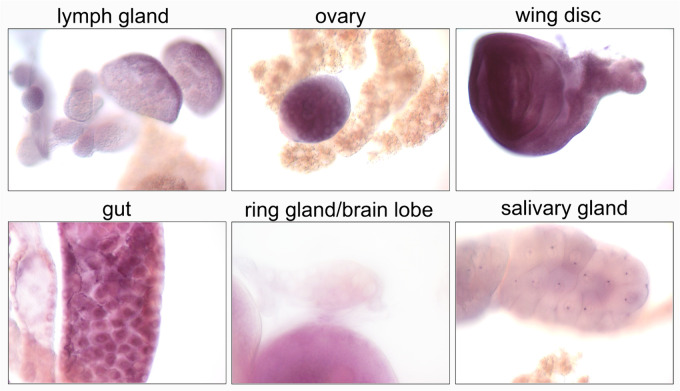
*trus* expresses in mitotic tissues. *In situ* hybridization with anti-sense RNA that hybridizes with *trus* mRNA reveals high level expression of *trus* in larval lymph gland, ovary, wing disc, gut, and brain lobe. Low expression is detected in ring and salivary glands.

We performed *in situ* hybridization with an anti-sense *trus* RNA probe to examine *trus* mRNA expression in 3rd instar wandering stage larval tissues. As shown in Fig 8, we detected *trus* expression at high levels in larval gut, ovary, brain lobe, wing disc, and lymph gland, where active cell proliferation is happening [63–66]. We also detected *trus* expression to a lesser extent in the salivary and prothoracic glands where cells are non-mitotic [67,68].

### Trus localizes to the cytoplasm and is exported from the nucleus in a CRM1/XPO1-dependent manner

**Fig 9:**
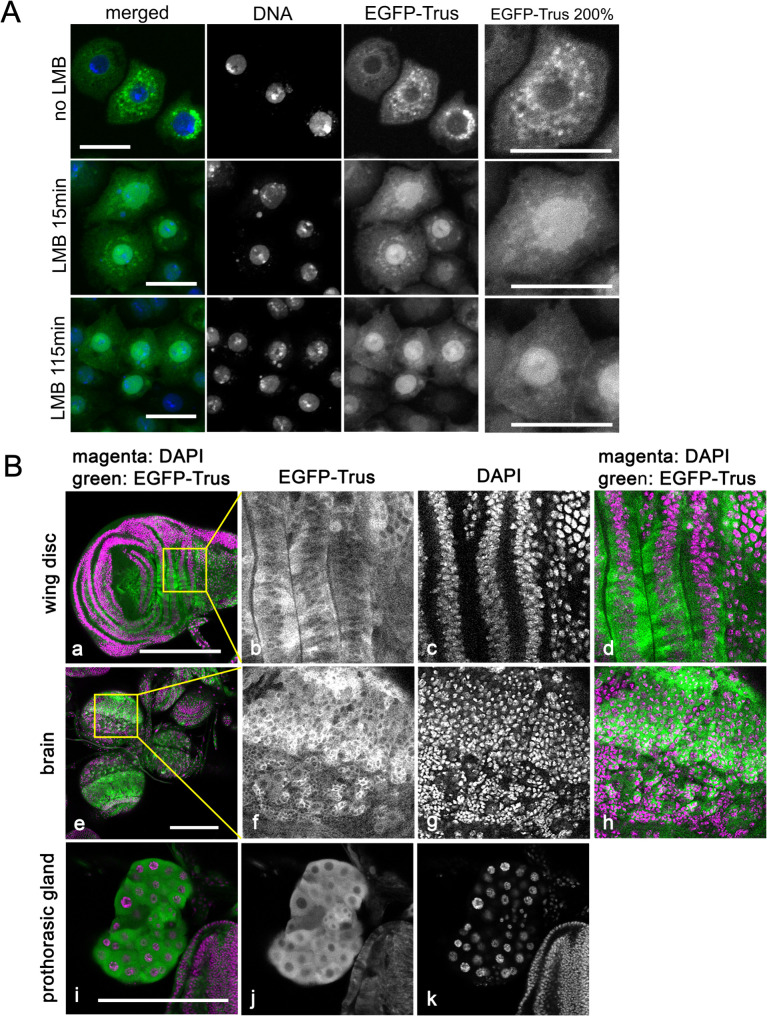
Trus localizes to the cytoplasm in cultured cells and *in vivo.* (A) *Drosophila* Trus localizes to the cytoplasm in S2 cells and shuttles between the nucleus and the cytoplasm in a CRM1/XPO1-dependent manner. Leptomycin B (LMB) is an inhibitor of CRM1/XPO1. Without LMB treatment (top row), EGFP-Trus expressed in S2 cells localizes primarily to the cytoplasm, and after treatment of the cells with LMB (middle and bottom rows), EGFP-Trus accumulates in the nucleus (LMB 15min, LMB 115min). Maximum intensity Z-projections are shown. Bar: 20μm. (B) *da-GAL4 induced UAS-EGFP-trus* expression in larval tissues. *da-GAL4* expression is ubiquitous in most of the tissues. EGFP-Trus primarily localizes in the cytoplasm of cells (b, f, and j). Representative images of a wing disc (a-d), brain (e-h), and prothoracic grand (i-k) are shown. (b-d) and (f-h) are magnified images of the area marked with a yellow square in a and e, respectively. Each image is a single Z slice from multiple Z series confocal scanning data. Scale bar: 200μm.

Landry-Voyer *et al.* [37] reported that PDCD2L, the human ortholog of Trus, primarily localizes to the cytoplasm but shuttles between the nucleus and the cytoplasm, and the exportation from the nucleus is dependent on CRM1/XPO1, the RanGTP-binding exportin, which recognizes a leucine-rich nuclear export signal (NES) sequence. They showed that while PDCD2L primarily localizes to the cytoplasm, inhibiting CRM1 with Leptomycin B (LMB) or mutating the predicted NES sequence leads to retention of the protein in the nucleus [37]. We investigated Trus protein localization in S2 cells and *in vivo* and found that *Drosophila* Trus protein localizes in the same way as the human PDCD2L. As shown in Fig 9A, EGFP-tagged Trus protein expressed in *Drosophila* S2 cells primarily localized to the cytoplasm (No LMB); however, after Leptomycin B treatment, EGFP-Trus became more concentrated in the nucleus, and longer LMB treatment was more effective (LMB 15min vs. LMB 115min), indicating that Trus shuttles between the nucleus and the cytoplasm in CRM1-dependent manner. We searched for a putative NES in Trus, based on NES consensus sequences reported in Kosugi *et al.*(2008) [69], and found at least 6 candidates in the loop region between the PDCD2_N and the PDCD2_C domains, which is much longer in Trus (273 a.a.) than human PDCD2L (132 a.a.) (shown in gray in Fig 7A and 7B). Further investigations by mutating the candidates individually or in combinations are necessary to determine which NES candidate(s) is the Trus NES(s).

To examine cellular localization of Trus *in vivo*, we over-expressed EGFP-tagged Trus using *UAS-EGFP-trus* driven by *da-GAL4.* EGFP-Trus was expressed ubiquitously in larval tissues, the over-expression did not affect viability of the flies and did not cause any defects in larval tissues. As shown in Fig 9B, EGFP-Trus localizes primarily to the cytoplasm within the epithelial cells in wing discs (Fig 9B, a–d), neurons in the brain (e-h), and cells in prothoracic gland (i-k). We confirmed that *da > UAS-EGFP-trus* rescues *trus* mutant lethality and developmental delay similar to *da > UAS-trus*, indicating that EGFP-Trus is functional (S3 Table). Our results suggest that the PDCD2L protein family including *Drosophila* Trus may work as a nucleus to cytoplasm shuttling transporter within the cell [29,37].

### Identifying the tissues that require Trus protein activity during development

**Fig 10:**
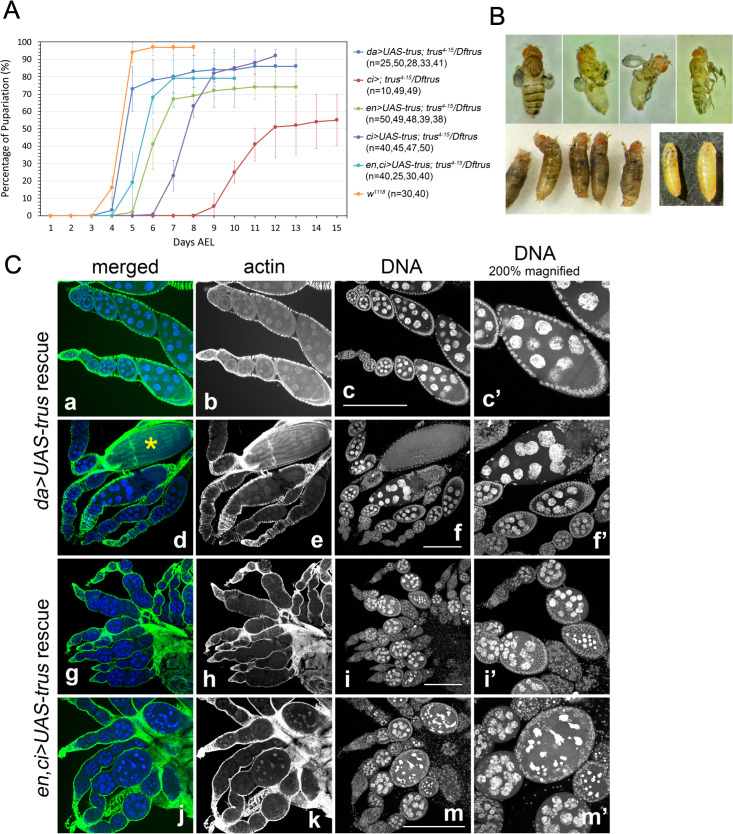
Rescue of lethality and developmental delay of the *trus* mutant can be achieved by Trus expression induced with specific drivers. (A) Developmental timing of *trus* mutants (*trus*^*4-15*^*/Dftrus*) with *trus* expression driven by various drivers display varying degrees of rescue. Compared to the *w*^*1118*^ control (orange), *da-Gal4* (dark blue) rescues the best, with *en-Gal4* and *ci-Gal4* (aqua), *en-Gal4* (green), and *ci-Gal4* (purple) showing progressively lower degrees of rescue. *ci-Gal4 without UAS-trus* (red) serves as the negative control. (B) Adult and pupal phenotypes of rescued lines. *ci > UAS-trus* rescued *trus*^*4-15*^*/Dftrus* pre-pupal lethality with significant defects in wings, halteres, legs, and bristles (upper panels). Many flies eclose only half-way from the pupal case and die (lower left). *trus*^*4-15*^*/Dftrus* mutants without the *UAS-trus* transgene arrest and die during the pre-pupal stage (lower right). (C) (a-f, c’, and f’) Ovariole phenotypes of rescued lines confirms that *da > UAS-trus* rescue of *trus*^*4-15*^*/Dftrus* mutant females. They are fertile and show no defects in oogenesis. The yellow star in d indicates a mature egg produced. (g-m, i’, and m’) *en,ci > UAS-trus* in combination with *trus*^*4-15*^*/Dftrus* mutants, while being able to rescue mutant lethality, give rise to females that are sterile. Their ovarioles produce no mature eggs because egg chambers degrade at mid-oogenesis. (green) Rhodamine-Phalloidin, (blue) DAPI. For each genotype, 10 ovaries from 10 individual female were dissected and all showed the similar phenotypes. Maximum intensity Z-projections are shown. Scale bar: 200μm.

To determine which tissue(s) require Trus function during development, we expressed Trus protein in *trus* mutant (*trus*^*4-15*^*/Dftrus*) animals from a *UAS-trus* transgene using various *GAL4* drivers and examined whether *trus* developmental delay or lethality was rescued (Fig 10A and Table 3). Trus expression with a ubiquitously expressed *daughterless-GAL4 (da-GAL4)* rescued both developmental delay and lethality of *trus*^*4-15*^*/Dftrus* animals (Fig 10A shown in blue). The eclosed adult flies had no defects and were fully fertile.

**Table 3:** Rescue summary of *trus*^*4-15*^*/Dftrus* developmental timing and lethality.

background	rescue by	pupariation days AEL	days rescued	lethality	places express the GAL4
*w* ^*1118*^	n/a*	5	n/a	viable	n/a
*trus* ^*4–15*^ */Dftrus*	n/a*	12	n/a	pre-pupal lethal	n/a
*trus* ^*4–15*^ */Dftrus*	da > UAS-trus**	5	7	89% eclosed^#^ w/o defects, fertile	Ubiquitous [70]
*trus* ^*4–15*^ */Dftrus*	en, ci > UAS-trus**	6	6	41% eclosed^#^ female sterile, oogenesis stops at stage5/6	*en* + *ci* *en* expression is stronger than *ci* when expressed together
*trus* ^*4–15*^ */Dftrus*	en > UAS-trus**	7	5	pre-pupal lethal	posterior half of imaginal discs, some cells in VNC ****
*trus* ^*4–15*^ */Dftrus*	ci > UAS-trus**	9	3	18% eclosed or half-eclosed^#^ w/severe defects in wings, halteres, legs	anterior half of imaginal discs, neuroepithelial cell lineages in optic lobes, low expression in some cells in VNC ****
*trus* ^*4–15*^ */Dftrus*	*nub > UAS-trus*	12	0	pre-pupal lethal	wing/haltere disc (pouch), some cells in central brain, low expression in some cells in leg discs ****
*trus* ^*4–15*^ */Dftrus*	*pdm2 > UAS-trus*	12	0	pre-pupal lethal	wing/haltere disc (pouch + upper posterior corner of hinge), a restricted area of leg discs, lamina cells in optic lobes, some cells in VNC ****
*trus* ^*4–15*^ */Dftrus*	*repo>UAS-trus*	12	0	pre-pupal lethal rescued mobility in wandering stage	pan-glial cells in brain [71]
*trus* ^*4–15*^ */Dftrus*	*elav>UAS-trus*	12	0	pre-pupal lethal	pan-neuronal cells in brain [71]
*trus* ^*4–15*^ */Dftrus*	*e22c>UAS-trus*	12	0	pre-pupal/pupal lethal some reach to pharate and die	epidermal cells [72], limited cells in CNS (our observation)
*trus* ^*4–15*^ */Dftrus*	*da>* UAS-dilp8 RNAi***	7	5	pre-pupal lethal	ubiquitous
*trus* ^*4–15*^ */Dftrus*	*da>* UAS-Xrp1 RNAi***	8	4	pre-pupal lethal^§^	ubiquitous

Trus protein was expressed in specific tissues with various *GAL4* drivers in *trus*^*4-15*^*/Dftrus* mutant animals. Graphs of pupariation timing of these rescue lines are shown in Fig 1B for *, in Fig 10A for **, and in Fig 5C for ***. ^#^ indicates the percentage of first instar larvae that ended up eclosing or half-eclosing as adult flies. *da: daughterless-GAL4*, *en: engrailed-GAL4, ci: cubitous interruptus-GAL4, nub: nubbin-GAL4, pdm2: pdm2-GAL4, repo: repo-GAL4, elav: elav-GAL4, e22c: e22c-GAL4.* **** Expressions are shown in S3 Fig. ^§^ Observed limited number of pupae/pharate adults that did not eclose depending on culture condition.

Trus expression driven by *ci-GAL4* or *en-GAL4* accelerated pupariation timing by up to 3 or 5 days, respectively, compared to the control, and a combination of *en-GAL4* and *ci-GAL4* accelerated pupariation timing by 6 days (Fig 10A). Since *en-GAL4* and *ci-GAL4* alone express in complementary halves of each imaginal disc, rescue of developmental timing with Trus expression using both drivers may work in a synergetic way (Fig 10A). We also found that *nub-GAL4* and *pdm2-GAL4*, which are strongly expressed in the wing pouch but not in a major part of the leg discs or the hinge/notum of wing discs, rescued neither the developmental delay nor lethality of the *trus* mutant (Table 3). This is not surprising since Dilp8 is likely to be strongly expressed in the notum and hinge area of wing discs, leg discs, and other discs in *trus* mutants when Trus is expressed only in wing pouch using *nub-GAL4* or *pdm2-GAL4*.

Importantly, while either *en-GAL4* and *ci-GAL4* driven Trus expression could individually rescue the developmental delay of *trus* mutants, the lethality of *trus* mutants was rescued only by *ci-GAL4*. Most of the *trus* mutant larvae with *ci > UAS-trus* pupated and reached the pharate stage. 18% of the animals eclosed or halfway-eclosed as adult flies with severe defects in legs and wings (Fig 10B and Table 3). We examined larval brain and wing disc for cell proliferation and tissue structures. As shown in S8A Fig, mitotic cells (PH3 foci) in brain lobes and VNC as well as the size and ring-structure of the brain lobes from *ci > UAS-trus; trus*^*4-15*^*/Dftrus* larvae look similar to those from *w*^*1118*^. Cell proliferation and tissue growth of wing/haltere/leg discs also appeared to be substantially rescued and were similar in size to those of *w*^*1118*^ larvae (S8B Fig). We noticed that PH3 foci are enriched in the half of each wing/haltere/leg disc, suggesting a cell-autonomous induction of mitosis as *ci-GAL* is expressed only in the anterior half of the discs therefore could induce Trus expression primally in the anterior half of the discs (S3 and S8B Figs).

As we have shown (S3 Fig), expression of *en-GAL4* and *ci-GAL4* complement each other in wing and leg discs (i-m and n-q in S3 Fig); however, their brain expression patterns are distinctly different. Notably, *ci-GAL4* is strongly expressed in a large number of cells in brain lobes including differentiating neural epithelia cells in optic lobes (n and o in S3 Fig), whereas *en-GAL4* expression in brain is limited to some cells in the VNC (i and j in S3 Fig). We speculate that it is this difference in central nervous system (CNS) expression of *ci-GAL4* vs *en-GAL4* that accounts for the rescue of *trus* lethality by one and not the other.

### Trus is essential for oogenesis

In our rescue experiments, we expressed Trus using a combination of *en-GAL* and *ci-GAL4* together. This double driver further accelerated pupariation timing up to 6 days (light blue in Fig 10A) and significantly rescued *trus* mutant lethality. 41% of 1st instar larvae eclosed as adult flies with no morphological defects (Table 3). However, we found that the eclosed females were completely sterile and laid no eggs. To determine when the block occurs in oogenesis, we dissected their ovaries and stained them with Rhodamine-Phalloidin and DAPI, As shown in Fig 10C, in *en,ci > UAS-trus* rescued ovaries, a normal number of egg chambers seemed to form, but nurse cell nuclei started to show abnormal morphologies at early stages and severe aggregation and fragmentation of the nuclear DNA by mid-oogenesis (stage 5/6) (Fig 10C, g-m, i’, and m’). Additionally, follicle cells that normally surround the developing nurse cells and oocyte allow the egg chamber to hold its structure and change its shape from spheroid to elliptical as seen in the control (Fig 10C, a–f, c’, and f’), also deteriorate and degrade by stage 5/6 in *en,ci > UAS-trus* rescue (see Fig 10C, i’ and m’ comparing to c’ and f’). Oogenesis seemed to arrest and egg chambers degraded completely after Stage5/6. Mature eggs always seen at the end of the egg chambers in the control ovaries (Fig 10C, d, an egg shown with yellow star) did not exist in ovaries derived from *en,ci > UAS-trus* rescued females. High-throughput transcriptome analyses have reported that both *engrailed* (*en*) and *cubitus interruptus* (*ci*) are not expressed or have very low expression in ovaries (http://flybase.org). Therefore, we infer that, although *en-GAL4* and *ci-GAL4* zygotically expressed *UAS-trus* and rescued *trus* mutant (*trus*^*4-15*^*/Dftrus*) lethality, the drivers did not express *UAS-trus* in ovaries, and thus did not rescue the Trus function that is essential for oogenesis.

### Trus is found in a complex with SOP/RpS2 and eEF1α1

To search for possible Trus interactors, we used the Tap-tagging system described by Veraksa *et al.* (2005) [73], which identifies at least transiently stable protein complexes. *Drosophila* Kc cultured cells expressing a Tap-Trus fusion were lysed, and interacting proteins were isolated by affinity chromatography against the tag. The final eluate from the affinity column had two major bands and one band of somewhat lower intensity that were not found in controls (S9 Fig). One of these bands was (as expected) Trus itself; this band assignment was verified both by Western blotting with anti-Trus antibody and by mass spectrometry. Using mass-spectrometry, the other major band (band 3 on S9 Fig) is identified as String of pearls (Sop), protein S2 of the 40S ribosomal subunit (RpS2) [30], while band 2 is identified as eukaryotic translation Elongation Factor 1 alpha 1 (eEF1α1: CG8280), a factor that plays a role in shuttling tRNAs to the ribosome during translation [74]. To the best of our knowledge eEF1α1 has not been previously associated with either PDCD2L or PDCD2 containing complexes. RpS2/Sop has been reported as a binding partner of Zfrp8 [42]. However, we did not find Zfrp8 in our Trus pulldown, suggesting Trus and Zfrp8 are not in a same protein complex supporting the idea that they play different roles in ribosome biogenesis and development [29].

## Discussion

In this report, we characterized the phenotypes associated with mutations in the gene encoding Trus, the *Drosophila* homolog of the putative vertebrate ribosome subunit assembly factor PDCD2L. The most apparent phenotypes are observed in larval mitotic tissues such as imaginal discs and the central nervous system, while no obvious defects were noted in many endocycling tissue such as epidermis, fat body, muscle, much of the gut, and the prothoracic gland. One exception is the ovaries where endocycling nurse cells exhibit altered morphology at stage 6, just before the egg chambers degenerate. To a first approximation, these phenotypes suggest that dividing cells are most sensitive to loss of Trus, an inference consistent with the need for a large cellular ribosomal content for rapid growth. It is not clear what aspect of the cell cycle is affected in *trus* mutant larvae, but it is intriguing to note that *trus* was also uncovered in a transcriptome profiling screen of wing imaginal discs and S2 cells as one of 63 genes that showed periodic transcription enriched at the G2 phase of the cell cycle in both cell types [75]. Furthermore, *RNAi*-mediated knockdown of *trus* in wing discs produced small abnormal adult wings, like what we report here, and cell cycle profiling of the *trusRNAi* wing disc cells using fluorescence-activated cell sorting (FACS) showed increased G2/M [75], implying a role for Trus in cell cycle control at the G2/M transition.

Interestingly, growth in many highly proliferative human cancer cell lines do not appear to require PDCD2L in contrast to PDCD2 [76] (https://depmap.org/portal/gene/PDCD2L), while in other lines it is highly overexpressed [29,41,77]. The reason for this dichtomy in cancer is not clear. However, in the context of normal development, PDCD2 and PDCD2L are both essential during embryonic stages with PDCD2L mouse knockout embryos being reabsorbed at mid-gestation while PDCD2 null embryos do not progress past the blastocyst/morula stages [40,41]. In *Drosophila*, both Zfrp8/PDCD2 and Trus/PDCD2L are essential for cell proliferation, oogenesis, embryogenesis, and larval development [31,43], suggesting that each paralog has a non-redundant function in each of these specific biological process. The preponderance of data supports a primary role for PDCD2L/Trus and its paralogous couple, vertebrate PDCD2/*Drosophila* Zfrp8, in ribosome biogenesis [29]. The ancient origin and high core conservation between the paralogs and the orthologs in diverse phyla are consistent with this idea (Figs 7 and S7) [39].

One key biochemical activity of both PDCD2/Zfrp8 and PDCD2L/Trus is binding to uS5/RPS2, a stable component of the small (40S) ribosomal subunit. A recent model proposes that this binding is important for the activities of these proteins as molecular chaperones for assembly and nuclear exporter of the pre-40S subunit [29,35–38]. Consistent with this view, human PDCD2L has been shown to contain a functional NES and shuttles between nucleus and cytoplasm in a CRM1/XPO1-dependent manner [37]. As shown in this report, *Drosophila* Trus/PDCD2L also physically binds to *Drosophila* Sop/RpS2, and while it primarily localizes to the cytoplasm, blocking the nuclear export machinery by Leptomycin B treatment, an inhibitor of CRM1/XPO1, leads to Trus retention in the nucleus (Fig 9). These similarities suggest that like its vertebrate counterpart, Trus likely plays a role in the maturation process of pre-40S subunit possibly acting as a protein adaptor for the CRM1/XPO1 in the pre-40S subunit nuclear exportation [29].

In addition, to these structural and biochemical similarities, certain aspects of the Trus loss-of-function phenotype also connect Trus function to ribosome biogenesis. Specifically, we find that *trus* hypomorphic alleles can produce rare escapers with phenotypes that include developmental delay, thin bristles and notched eyes which are the primary characteristics of *Minute* mutants (S1 Fig). The *Minute* phenotype is produced by mutations in many ribosomal protein subunits. However, in the *Minute* case, the phenotype is observed in heterozygotes (*M/+*), while with *trus,* heterozygotes are completely normal with respect to bristles, eye morphology and developmental timing. It is only when there is presumably less than 50% gene product that we see rare escapers with the classic *Minute* phenotype. We believe the probable explanation is simply that ribosomal subunits are required stoichiometrically as structural components of ribosomes, while Trus likely provides a temporal adaptor/transporter function to pre-40S subunits and can be recycled/shuttled back to the nucleus, making it less sensitive to heterozygosity.

Interestingly, the original *sop/uS5* mutants were also hypomorphs and showed classic *Minute* phenotypes [30]. It is also noteworthy that the *sop*^*P*^ hypomorph is female sterile and blocks oogenesis at about stage 5 at which time nurse cell nuclear morphology becomes aberrant and degeneration occurs. This phenotype is strikingly similar to what we observe in *en,ci > UAS-trus* rescued animals in which oogenesis is defective. However, we also observe that *en,ci > UAS-trus* rescued egg chambers show severe deterioration of follicle epithelia by stage 5, which is earlier than *sop*^*P*^ mutants (Fig 8C) [30]. *Zfrp8* escapers are also reported to be female sterile [31]. Additional studies employing somatic and germline clones revealed a requirement of *Zfrp8* for proliferation of both germline stem cells and follicle stem cells. Germline clones progressed as far as stage 8 followed by degeneration [43], again similar to *sop* hypomorph alleles and the *en,ci > UAS-trus* rescue egg chambers.

Given that Trus and Zfrp8 are paralogs and that their vertebrate counterparts appear to act as chaperone/adaptors during assembly and translocation of pre-40S subunit in and out of the nucleolus, it is perhaps not so surprising that they exhibit several common phenotypes, including lethality during all larval stages, developmental delay during the third instar, and oogenesis defects under hypomorphic conditions. Certain aspects of the null mutant phenotypes, however, are not identical. Minakhina *et al.* (2007) [31] noted that they obtained up to 5% escapers from *Zfrp8* null alleles while *trus* null larvae progress to prepupae, but none make it to adults. These differences, and the general polyphasic lethality we observe, could simply represent different degrees of perdurance of maternally supplied RNA or protein for the different genes. Another notable difference is that *zfrp8* loss-of-function alleles produce overproliferated lymph glands, perhaps due to an altered cell cycle [31]. In *trus* null mutants, the lymph gland is not particularly enlarged. However, the putative antimorphic/neomorphic *trus*^*1*^ allele do exhibit enlarged lymph glands (S10 Fig). In this case, we envision that an N-terminally truncated Trus, that might arise from aberrant translational starts at downstream methionine codons, could act as a dominant negative form of Trus. Specifically, since PDCD2 and PDCD2L appear to occupy the same binding site on uS5/RPS2, then an N-terminally truncated Trus might fully or partially block Zfrp8 activity leading to lymph gland overgrowth

One other notable difference between Trus and Zfrp8 concerns their interaction partners. Mass spec analysis of proteins pulled down by an embryonically expressed N-terminally TAP tagged Zfrp8 identified 31 proteins including RPS2 [44] which we also identified as an interacting partner of N-terminally TAP-tagged Trus. Interestingly, despite the fact that both Trus and Zfrp8 share the RpS2/Sop as an interacting partner, Trus and Zfrp8 were not found in a common protein complex (this work) [44]. In addition, eEF1α1, which was a prominent band in our Trus pull down (S9 Fig), was not identified as a component of Zfrp8 complexes consistent with the idea that each paralog likely has its own complement of unique interacting factors which then provides each complex with the potential for a distinct non-overlapping biological activity.

A key common phenotype of *trus, Zfrp8*, *M/+* and another ribosomal biogenesis component mutant known as *Noc1* is developmental delay during the third instar larval stage [4,28,31]. The cause of this phenotype has not been examined for Z*frp8* mutants but has been studied in *Minute* and *Noc1* mutants where it has been shown that subunit haploinsufficiency or improper ribosomal biogenesis triggers activation of the Xrp1 stress response pathway. In the case of *M/ + *mutants, activation of the Xrp1 transcription factor requires input from RpS12, a ribosomal component, that appears to act as a sensor of ribosomal subunit concentration via an unknown mechanism, while in *Noc1,* the Eiger-JNK pathway is activated which can also induce Xrp1 [16,28]. Interestingly, Kiparaki *et al.* (2022) have reported that depleting various translation initiation, elongation, or termination factors including eEF1α1, which we identified as a binding partner of Trus, also induces Xrp1 expression leading to eIF2α phosphorylation, reduced translation, and cell competition [78]. Thus, Trus protein may disrupt the localization or function of eEF1α1, leading to an overall translational reduction.

Once induced, Xrp1 can lead to a wide range of activities including blocks in translation and proteasome flux, activation of DNA repair and antioxidant genes, and, most relevant for this discussion, upregulation of *dilp8* in imaginal discs [8,15,27]. Dilp8 itself is a systemic stress signal that slows development by binding to a subset of Lgr3 positive neurons in the brain. These neurons synapse with the PG neurons that produce several developmental timing signals including PTTH, a neuropeptide that induces a rise in ecdysone production just prior to metamorphosis. Activation of the Lgr3 neurons by Dilp8 inhibits the rise in PTTH and thereby delays ecdysone production resulting in a pronounced developmental lag during the third instar stage [56,79].

As we demonstrate in this report, Dilp8 expression is strongly activated by loss of Trus and whole animal knockdown of either Xrp1 or Dilp8 ameliorates the developmental delay (Fig 5), and at least for Xrp1 knockdown, the reduction in brain and wing disc size (S5 Fig). Since ecdysone feeding also largely rescues the Xrp1-Dilp8 developmental delay [55] (Fig 6), the simplest explanation for the brain and disc size rescue is that knockdown of Xrp1 (S5 Fig), and to some extent Dilp8 (S6 Fig), restore basal ecdysone production to near normal levels and thereby rescue brain and disc proliferation which are both known to be controlled by basal ecdysone levels [15,54]. The difference in the degree of rescue between Xrp1 and Dilp8 knockdown could simply reflect differences in knockdown efficiencies although it is also possible that some other non-Dilp8 signal, could contribute to the developmental delay and cell proliferation phenotypes.

Another consideration with respect to developmental delay is that *trus* larvae never make it past the pre-pupal stage, whereas *Ptth* mutants are viable as are *M/ +* mutants that also exhibit Dilp8-induced developmental delay [22]. This suggests that there are likely additional hormone imbalances in *trus* mutants. One possibility is that high levels of Trus are needed in the prothoracic gland (PG) itself to enhance production of the 20E biosynthetic enzymes which are necessary for producing the large pre-metamorphosis inducing 20E peak. It is interesting to note that reduction in Noc1 has been shown to delay development both by inducing Dilp8 expression in imaginal discs and through direct effects in the prothoracic gland on ecdysone production [28].

Although the causative mechanism behind the developmental delay, i.e., Xrp1-Dilp8 induction in *trus* mutants, is quite similar to that seen in *M/+* and *Noc1* mutants, once again there are some notable differences among the three. First, while we observe extensive proliferation defects in the CNS of *trus* mutants, such defects have not been reported for *M/+* or *Noc1* mutants. For *M/ + *, this could simply be a dosage effect since the *trus* mutant is a homozygous null and may produce a more compromised cell than a *M/ + *mutant which is a heterozygous knockdown of an individual RPS. For *Noc1,* null mutants were not examined and knockdown of *Noc1* in neurons did not produce a phenotype [28]. It would be interesting to determine if knockdown of an RPS or Noc1 in neuroblasts during larval stages produces a small brain phenotype like what we observe in *trus* mutants.

A second difference between *trus* and these other two types of alterations in ribosome biogenesis is induction of apoptosis through activation of cell competition. Heterozygosity for an RPS in *M/ +* mutants results in pronounced apoptosis in imaginal discs and a simultaneous compensatory proliferation that allows the imaginal disc to reach its normal size and shape and to produce a normal appendage [22]. Inhibition of *M/ +* induced apoptosis by expression of baculovirus p35 causes development of abnormal wing morphology indicating that apoptosis and the compensatory proliferation must be balanced to produce a normal structure. The apoptosis effect appears to be mediated to a large extend by Wg-induced cell competition [22]. RNAi knockdown of *Noc1* in wing discs also produces substantial apoptosis through both Eiger-JNK and Xrp1 induction [28]. In the case of *trus* mutants, we observe decreased, rather than increased, proliferation as seen in *M/ +* mutants and no significant apoptosis within the discs or brain, as monitored by TUNEL assay or anti-cleaved caspase 3 staining. It seems that Xrp1 induction does not cause apoptosis in discs fully mutant for *trus* where there would not be any cell competition. Surprisingly, we also do not see apoptosis when we use *trus* RNAi to knockdown Trus in only a portion of the disc (i.e., *nub-GAL4 > trusRNAi*, *en-GAL4 > trusRNAi*). Additional studies will be required to address these differences.

In summary, our results highlight a role for the highly conserved ribosomal assembly protein Trus/PDCD2L in the control of mitotic proliferation, tissue growth, and oogenesis during *Drosophila* development. When the levels of this protein are reduced by mutation, the Xrp1-Dilp8 stress signaling pathway is triggered which slows development through inhibition of ecdysone production in an attempt to correct the cell proliferation and tissue growth imbalance.

## Materials and methods

### Fly lines

Detailed genotype and sources of fly stocks that were used in this study are listed in S1 Table. Flies were reared on standard cornmeal fly medium at either 18, 25°C, or room temperature depending on the purpose.

### Production of *trus* CRISPR/Cas9 mutants

*trus* mutants were produced using the CRISPR/Cas9 system. Two target sequences in the *trus* gene (CG5333) that are unlikely to have off-target binding were identified using CRISPR Optimal Target Finder (http://targetfinder.flycrispr.neuro.brown.edu/) [80]. Sense and antisense double strands of the targets A and B (see S2 Table) were cloned into *pU6-BbsI-chiRNA* (Addgene #45946) [46] for production of single stranded guide RNAs (sgRNAs) and were co-injected into vas-Cas9 embryos (BDSC_51323) by BestGene Inc. (Chino Hills CA). Single G0 adults were crossed to *w*^*1118*^ and then 10 F1 males were crossed to *w*^*-*^*; Bl/CyOGFP; TM2/TM6B P[Dfd-GMR-nvYPF]SbTb.* From the established lines balanced over *TM6B P[Dfd-GMR-nvYPF]SbTb*, homozygous lethal lines were selected as *trus* null mutant candidates. Genomic DNA from balanced adults or homozygous larvae from each candidate line was prepared [81] and a 1.7kb *trus* genomic region was amplified by PCR (Expand High Fidelity PCR System, Millipore Sigma, Burlington, MA) with two primers, Trus2_for and Trus1_rev (S2 Table). Of the 5 pupal lethal lines from G0 #35, 4 had the same deletion of 1184 bp. Line 35–2 was selected and is hereafter called *trus*^*35-2*^. Of the 5 pupal lethal lines from G0 #4, 2 had the same 1 bp deletion at target A and were designed *trus*^*4-15*^. Genomic DNA from *trus*^*1*^*/ TM6B P[Dfd-GMR-nvYPF]SbTb* adults was also prepared and sequenced as described above with Trus1_for, Trus3_rev, TrusB_for, and TrusC_for primers.

### Trus antibody production

Full length Trus protein N-terminally tagged with 6xHis-EGFP (His-EGFP-Trus) was expressed in Sf9 cultured insect cells using the Bac-to-Bac Baculovirus Expression System (Thermo Fisher Scientific, Waltham MA). The *trus* coding sequence, codon-optimized for insect cell expression and N-terminally tagged with 6xHis-EGFP, was cloned into pFastBac1 (*pFB-His-EGFP- HRV3C -trus*) using NEBuilder High-Fidelity DNA Assembly (New England Biolabs, Ipswich MA). A HRV3C cleavage target sequence (3C) was inserted between EGFP and Trus. The plasmid was transformed into DH10Bac E. coli. cells and recombinant bacmids were isolated. Sf9 cells were seeded in a 6-well plate and transfected with the His-EGFP- HRV3C -Trus bacmid using CellFectinII (Thermo Fischer Scientific). The transfected cells were cultured at 28°C for 72 hrs. The supernatant (P0 virus stock) was collected and subsequently used to infect 10mL Sf9 cells to produce a P1 virus stock; the P1 was used to produce a 100mL P2 virus stock, and the P2 was used to produce a 300mL P3 virus stock. Virus infection of each amplification stage was confirmed under an optical microscope and His-EGFP-3C -Trus protein production was monitored by EGFP expression under a fluorescent microscope. Virus titer was measured for the P3 virus stock by Expression Systems (Davis CA) and used for subsequent His-EGFP-Trus protein expression. His-EGFP-3C-Trus recombinant protein was expressed in 2L of Sf9 cells, and the cell lysate was applied to a TALON CellThru metal affinity resin column (Takada Bio USA Inc., San Jose CA). The column was washed, and His-EGFP-3C-Trus protein was eluted with 150mM imidazole. The protein elution was monitored with EGFP fluorescence, and EGFP- positive fractions were collected and combined. The combined elution was incubated with HisGST-HRV3C protease to cut the His-EGFP tag at the 3C site, concentrated using Amicon Ultra-15 (MilliporeSigma, Burlington, MA), and incubated overnight at 4°C. It was then buffer-exchanged to lower imidazole concentration to 10mM using a Sephadex G-25/PD-10 desalting column (Cytiva, Marlborough MA) and combined with HisPur Ni-NTA resin (Thermo Fisher Scientific) and incubated at 4°C for 3 hours. The protein-resin solution was centrifuged, and the supernatant that contained Trus protein was collected, concentrated with Amicon Ultra-15, and loaded onto a Superdex-200 gel filtration column (MilliporeSigma). Trus protein containing fractions were combined, buffer-exchanged to Phosphate Buffer Saline (PBS, pH7.4) and concentrated. This concentrated purified Trus fraction was used to inject two rabbits to produce polyclonal anti-Trus antibody by Thermo Fisher Scientific. Anti-Trus antibody (AB2866) was affinity-purified from serum obtained from one of the rabbits with bacterially expressed recombinant Trus protein using AminoLink Plus Coupling Resin (Thermo Fisher Scientific) and was used for Western blotting (WB) at 1:2000.

### Other antibodies and cell staining reagents

Phospho-histone H3 (Ser10) antibody (Cell Signaling Technology, Danvers MA) was used for IF at 1:1000 and α-Tubulin antibody (DM1A) (MilliporeSigma, T9026) was used for WB at 1:5000. Alexa 488-Goat anti-Rabbit IgG (H + L) and Alexa 555-Goat anti-Mouse IgG (H + L) Secondary Antibodies were used at 1:500–1:1000 for IF (Cell Signaling Technology). DyLight 680-Goat anti-Mouse IgG (H + L) and DyLight 800-Goat anti-Rabbit IgG(H + L) Secondary Antibodies were used at 1:10000 for WB (Thermo Fisher Scientific). For DNA staining, 4’,6-diamidino-2-phenylindole (DAPI) was used at 1.25mg/ml (MilliporeSigma), for F-actin staining, Rhodamine-Phalloidin was used (Thermo Fisher Scientific).

### Western blotting

Wandering 3rd instar larvae of *w*^*1118*^ and *trus* mutants were collected and homogenized in SDS-PAGE sample buffer, boiled, and centrifuged to remove debris. Samples were run on 4–12% NuPAGE Bis-Tris Mini Protein gels (Thermo Fisher Scientific) together with the purified recombinant Trus protein fraction and the separated proteins were transferred to Polyvinylidene fluoride (PVDF) membrane. The membrane was blocked with x1/10 1xPBS1%Casein Blocker (BIO-RAD, Hercules CA), probed with purified rabbit anti-Trus antibody (this study) and mouse anti-α-Tubulin antibody (MilliporeSigma, T9026), washed, and then detected with DyLight secondary antibodies (Thermo Fisher Scientific). Finally, the membrane was washed and scanned with the Odyssey M imaging system (LICORbio, Lincoln NE).

### Immuno-fluorescence staining, imaging, and image analysis

Third instar wandering stage larvae were roughly, turned inside-out and removed most of the gut and the fat tissues removed in PBS, fixed with 3.2% EM grade Paraformaldehyde (Electron Microscopy Sciences, Hatfield PA) in PBS for 25 minutes, washed multiple times with PBST (0.3% Triton-X100, 1x PBS) on rotary shaker at room temperature. They were then incubated with primary antibody solution overnight at 4°C, washed with PBST and incubated with Alexa Fluor conjugated secondary antibody solution (Thermo Fisher Scientific, Waltham MA) overnight at 4°C. Next day, they were rinsed with PBST, incubated with DAPI and/or Rhodamine-phalloidin for 5min. at RT, washed again multiple times with PBST. Brain and imaginal discs were dissected and mounted on a slide glass with anti-fading 1,4-Diazabicyclo[2.2.2]octane (DABCO) containing glycerin/PBS solution. Images were acquired with a Zeiss LSM 710 laser scanning confocal microscope using Zen imaging software (Zeiss, Jena Germany). To scan *Drosophila* tissues, 20x Plan-Apochromat/0.8 objective was used. An Argon laser, a HeNe laser, or a 405nm Diode laser were used to detect Alexa Fluor 488 and EGFP, Alexa Fluor 555 and mRFP, or DAPI, respectively. Typically, 25–40 Z slices with 1.20μm interval covering the entire thickness/stack of brain or disc (pressed to 25–45μm) were scanned and ImageJ/Fiji [82] (https://imagej.net/software/fiji/) was used to construct a maximum intensity Z-projection from the Z slices. Further image analysis and quantifications were performed using ImageJ/Fiji as well. Based on the image analyses, box-and-whisker plots were generated and ANOVA analysis followed by Dunnett’s test to compare each mutant to qwild-type (w1118) were preformed using Prism10 (Graph Pad software, Inc., Boston, MA.

### *In situ* hybridization

To make the *trus* antisense *in situ* probe, the *pFLC-1-trus cDNA* clone (BDGP RE69372) was linearized with Eco53K1 and transcribed using DIG RNA Labeling Mix with T3 RNA polymerase (Roche, Indianapolis IN) according to manufacturer’s instructions. Wandering larvae of *w*^*1118*^ and *trus*^*1*^ were collected, dissected, and fixed. Hybridization using the digoxigenin-labeled antisense *trus* RNA probe and detection were performed as described previously [83,84].

### Generating *UAS-trus* and *UAS-EGFP-trus* transgenics

To generate *UAS-trus* and *UAS-EGFP-trus* transgenics, the Trus coding sequence from an EST plasmid (Berkeley Drosophila Genome Project: RE69372) [85] and EGFP coding sequence from pEGFP-c1 (Clontech) were cloned into *pUAST.attB* [86] using NEBuilder HiFi DNA Assembly (New England Biolabs) and injected into embryos derived from a cross between flies with the *attP* recombination site inserted at 2L-VK00037 (BDSC_9752) and the *dPhiC31* integrase source (PhiC31 integrase source; BDSC_40161) for phiC31-mediated site-specific recombination [86] and screened for *w+* transgenics that integrated *UAS-trus* or *UAS-EGFP-trus* (BestGene Inc., Chino Hills CA).

### Developmental timing assay

*trus*^*4-15*^, *trus*^*35-2*^, and *Dftrus* chromosomes were balanced over *TM6B* *P[Dfd-GMR-nvYPF]Sb*. As the *Dfd-GMR-nvYPF* fluorescent marker shows distinctive eye expression from embryonic stage 13 to adulthood, it is easy to score at the early larval stage [87]. These flies were crossed in different combinations. Embryos were collected on apple juice plates for 12–24 hours. 30–50 1st instar larvae (24–36 hours AEL) were screened for *Dfd-GMR-nvYFP* negative under a fluorescent dissecting microscope to obtain 1st instar larvae of *trus*^*4-15*^*/Dftrus, trus*^*35-2*^*/Dftrus*, *trus*^*4-15*^*/ trus*^*35-2*^, *trus*^*4-15*^ homozygous, or *trus*^*35-2*^ homozygous and transferred to a standard cornmeal-agar fly medium in a 60mm culture dish. Another 60mm culture dish with a 20mm hole in the center covered with nylon mesh was placed as a lid and tightly taped together. 3–4 plates (total of at least 150 larvae) for each genotype were prepared and placed in humidified chambers at 25°C. Each plate was observed under a dissecting microscope throughout the subsequent development, and pupariation, pupation, and adult eclosion were assessed once every 24 hours for 10–18 days.

### Ecdysone feeding assay

α-Ecdysone (MilliporeSigma E9004) and 20-Hydroxyecdysone (MilliporeSigma H5142) were dissolved in ethanol at 10mg/mL and used as stock solutions. 50μL of either (1) water, (2) ethanol, (3) α-Ecdysone stock solution, or (4) 20-Hydroxyecdysone stock solution were mixed with 2g of mashed standard cornmeal/agar fly medium and placed in the center of 60mm plastic culture dish. The supplemented fly medium was air-dried for 2–3 hours to allow ethanol to evaporate. Cages were set up for *w*^*1118*^ and a cross of *trus*^*4-15*^*/TM6SbTbHuYFP* x *Dftrus/TM6SbTbHuYFP,* and embryos were collected on apple juice agar plates for 12–24 hours. 50 first instar larvae of *w*^*1118*^ or *trus* mutant (nvYFP negative) were transferred to the prepared supplemented or control media for following developmental timing (Fig 6A).

### Trus rescue experiment

To perform Trus expression rescue experiments, the *UAS-trus* transgene on the second chromosome was combined with *Dftrus (Df(3R)BSC847)* on the third chromosome that was balanced with *TM6B P[Dfd-GMR-nvYPF] Sb* [87]. On the other hand, each of the *GAL4* lines were combined with the *trus*^*4-15*^ allele that was maintained over *TM6B P[Dfd-GMR-nvYPF] Sb* (ex. *w*^*1118*^; ***-GAL4; trus^*4-15*^*/ TM6B P[Dfd-GMR-nvYPF]SbTb or w*^*1118*^;; ***-GAL4, trus^*4-15*^*/ TM6B P[Dfd-GMR-nvYPF] Sb*). Then, *w*^*1118*^*; UAS-trus; Dftrus/ TM6B P[Dfd-GMR-nvYPF] Sb* flies were crossed with each of the *GAL4* fly line bearing the *trus* ^*4-15*^ allele. Embryos were collected on an apple juice plate, 1st instar larvae without the nvYFP marker (***-GAL4 > UAS-trus in the *trus*^*4-15*^*/Dftrus* background) were collected, transferred to regular cornmeal fly food in a 60mm dish and monitoring for developmental timing.

### RNAi

To knockdown *dilp8* or *Xrp1* in a *trus* mutant, *UAS-dilp8RNAi* or *UAS-Xrp1RNAi* on the second chromosome was combined with *Dftrus* on *TM6B P[Dfd-GMR-nvYPF]SbTb* balancer chromosome to make *w*^*1118*^*; UAS-dilp8RNAi; Dftrus/ TM6B P[Dfd-GMR-nvYPF]SbTb* and *w*^*1118*^*; UAS-Xrp1RNAi; Dftrus/ TM6B P[Dfd-GMR-nvYPF]SbTb*. Those flies were crossed with *w*^*1118*^*;; da-GAL4, trus*^*4-15*^*/ TM6B P[Dfd-GMR-nvYPF]SbTb* to ubiquitously induce the RNAi. 1st instar larvae negative for nvYFP were transferred to 60mm culture dishes that contain regular fly food at 24–36 hours AEL for following developmental timing. To knockdown *trus*, each *GAL4* driver line was crossed with a fly line bearing the *UAS-trusRNAi* and *UAS-dicer2* transgenes. 250–400 1st instar larvae of *nub-GAL4 > UAS-trusRNAi*, *pdm2-GAL4 > UAS-trusRNAi*, or *en-GAL4 > UAS-trusRNAi* were collected along with controls (*w*^*1118*^ and *UAS-trusRNAi* without a driver) and monitored for developmental timing.

### Trus localization in S2 cells

To express N-terminal EGFP-tagged Trus protein in S2 cells, the 6xHis-EGFP-HRV3C and Trus coding sequences (BDGP: RE69372) were assembled together on the *pMT-puro* vector using NEBuilder HiFi DNA Assembly (New England Biolabs). EGFP-tagged Trus expression induced with *da-GAL4 in vivo* completely rescued the *trus* mutant (*trus*^*4-15*^*/Dftrus*) phenotypes similarly to un-tagged Trus (S3 Table), indicating that N-terminal EGFP tagging does not disrupt Trus function. S2 cells were transfected with the *pMT-HisEGFP-Trus-puro* plasmid and a stably transfected cell line was established by a few weeks of puromycin selection. The stably transfected S2 cells were cultured in 12-well plates and induced EGFP-Trus expression was induced with 0.7mM CuSO4. At day3, the cells were suspended in flesh media and a Concanavalin A-coated cover glass was placed in each well and left for 30 min. The wells were treated with or without 10nM Leptomycin B (InvivoGen, San Diego CA). The cover glasses were removed from the well at either 15 mins. or 115 mins., and S2 cells on each cover glass were immediately fixed with 3.2% paraformaldehyde/PBS, washed, stained with DAPI, and rinsed. Each cover glass was mounted on a slide glass facing up-side-down with DABCO/glycerol/PBS anti-fade mounting media. Edges of the cover glass were sealed with nail polish. EGFP and DAPI signals were scanned with the LSM710 laser confocal scanning microscope (Zeiss).

### Purification of protein complexes containing Trus

The coding sequence of *trus* from cDNA clone RE69372 was cloned into *pMK33-NTAP* [73], and the resulting constructs were stably transfected into *Drosophila* Kc cells using CellFectin (Thermo Fischer Scientific). The TAP-Trus fusion protein was assayed on Western blots using HRP-conjugated anti-Protein A antibody (Rockland, Gilbertsville PA) and by immunofluorescence of fixed cells using goat anti-Protein A antibody at 1:1000 followed by TRITC-conjugated anti-goat antibody (Jackson Laboratories, Bar Harbor ME) at a dilution of 1:500. Protein complexes from one liter of TAP- Trus-expressing cells were isolated following Puig *et al.*, 2001 [88], using a lysis buffer for making *Drosophila* extracts [73]. After purification using IgG-Sepharose and Calmodulin-Sepharose beads (Thermo Fisher Scientific - Invitrogen), the final eluate was precipitated with trichloroacetic acid (TCA), resolubilized in Laemmli sample buffer (BIO-RAD) and subjected to SDS-PAGE. Bands were excised, trypsinized and analyzed by MALDI (Matrix-assisted laser desorption/ionization) mass spectrometry (Cornell Institute of Biotechnology) [89].

## Supporting information

S1 Fig t*rus*^*1*^ mutant shows ‘Minute’ syndrome phenotype. *trus*^*1*^ mutant (*trus*^*1*^*/Dftrus*) larvae delay development, and most of them are pre-pupal lethal. Rare escaper adults (~1/ vial) eclose showing rough/notched eyes and thin/short bristles which resemble the haplo-insufficiency ‘Minute’ syndrome that is often observed in flies carrying a mutation in one of the genes encoding ribosomal proteins. Wild-type eye and bristles phenotypes are shown in left.
(TIF)

S2 Fig t*rus*^*1*^*, trus*^*4-15*^*, trus*^*35-2*^ mutants lack full-length Trus protein. Western blot analysis of third instar larval lysate of *trus* homozygotes and heterozygotes over a balancer chromosome (*TM6B P[Dfd-GMR-nvYPF] Sb*). Molecular weight standards are indicated at the left in kDa. Recombinant full-length Trus protein that was purified from Sf9 cells and used as antigen for the anti-Trus antibody production was loaded on lane 2. Larval lysates were loaded from lane 3–9 and genotypes are shown on the top. Green shows signals detected with anti-Trus primary antibody and then DyLight 800-anti-rabbit IgG secondary antibody (Thermo Fisher Scientific). Red shows signals detected with anti-α-Tubulin antibody (DM1A) (Sigma-Aldrich T9026) and DyLight 680-anti-mouse IgG secondary antibody. Full length Trus protein and α-Tubulin (loading control) are indicated by arrows at the right.
(TIF)

S3 Fig Wing disc GAL4 driver expressions. *UAS-RedStinger* (shown in magenta) was induced with either *nub-GAL4* (a-d), *pdm2-GAL4* (e-h), *en-GAL4* (i-m), *ci-GAL4* (n-q), or *en-GAL4* plus *ci-GAL4* (r-u) drivers. DAPI staining (green) and RedStinger (magenta) are shown in first (a, e, i, n, and r) and third (c, g, k, q, and t) rows. RedStinger (white) is displayed in the second (b, f, j, o, and s) and fourth (d, h, m, q, and u) rows. For each genotype, 10 larvae were dissected, and representative images are shown. Each image shows the maximum intensity Z-projection. Scale bar: 200μm.
(TIF)

S4 Fig Overexpression of baculovirus p35 in *trus* mutant. Overexpression of baculovirus p35, an apoptosis inhibitor, did not rescue the defects in tissue growth and cell proliferation in *trus* mutant larvae. Representative images of brain (top two rows) and wing disc (bottom two rows) from third instar wandering larvae of *w*^*1118*^ (left), *trus*^*4-15*^*/Dftrus* (middle), and *da-GAL4 > UAS-p35* in *trus*^*4-15*^*/Dftrus* (right) are shown. Fixed tissues were stained with DAPI and anti-PH3 antibody. Brain and wing discs from p35 expressed *trus*^*4-15*^*/Dftrus* larvae are smaller and show significantly less mitotic cells (PH3 foci) compared to brain and wing discs from *w*^*1118*^ larvae, similar to brain and wing disc from *trus*^*4-15*^*/Dftrus* larvae without p35 expression, indicating that ubiquitous expression of p35 in *trus* mutant did not rescue the small brain/wing disc phenotype and cell proliferation defects. 16 larvae of *da-GAL4 > UAS-p35; trus*^*4-15*^*/Dftrus* were dissected, and all showed the defects similar to *trus*^*4-15*^*/Dftrus* mutant. Pupariation of p35 expressed *trus*^*4-15*^*/Dftrus* larvae occurred between 12–15 days AEL similar to *trus*^*4-15*^*/Dftrus* and the pre-pupae were 100% lethal. Each image shows the maximum intensity Z-projection. Scale bar: 200μm.
(TIF)

S5 Fig *Xrp1 RNAi* induced with *da-GAL4* in *trus* mutant. *Xrp1 RNAi* partially rescues cell proliferation defects in *trus* mutant brain and wing disc. (A) Representative images of *trus*^*4-15*^*/Dftrus* mutant brains that were induced *Xrp1 RNAi* with *da-GAL4*. Anti-PH3 foci number and brain size in *trus*^*4-15*^*/Dftrus* mutants were significantly increased by *Xrp1 RNAi* compared to the *trus*^*4-15*^*/Dftrus* mutants. (B) Representative images of *trus*^*4-15*^*/Dftrus* mutant wing/haltere/leg discs that were induced *Xrp1 RNAi* with *da-GAL4*. Anti-PH3 foci number and size of wing/haltere/leg discs were moderately increased compared to the *trus*^*4-15*^*/Dftrus* mutants. 16 larvae were dissected, and representative images are shown. Each image shows the maximum intensity Z-projection. Scale bar: 200μm.
(TIF)

S6 Fig *dilp8 RNAi* induced with *da-GAL4* in *trus* mutant *dilp8 RNAi* partially rescues cell proliferation defects in *trus* mutant brain. (A) Representative images of *trus*^*4-15*^*/Dftrus* mutant brains that were induced *Dilp8 RNAi* with *da-GAL4*. Anti-PH3 foci number and brain size in *trus*^*4-15*^*/Dftrus* mutants were moderately increased compared to the original *trus*^*4-15*^*/Dftrus* mutants by *Dilp8 RNAi*, but still less than *w*^*1118*^ level. (B) Representative images of *trus*^*4-15*^*/Dftrus* mutant wing/haltere/leg discs that were induced *Dilp8 RNAi* with *da-GAL4*. Anti-PH3 foci number and size of wing discs was not rescued at all compared to the *trus*^*4-15*^*/Dftrus* mutants. 16 larvae were dissected, and representative images are shown. Each image shows the maximum intensity Z-projection. Scale bar: 200μm.
(TIF)

S7 Fig Common core structural module of Trus, its orthologs, and Zfrp8. (A) AlphaFold structure of *Drosophila melanogaster* Zfrp8 (Accession number: Q9W1A3) with domains PDCD2_N (green and light blue), PDCD2_C (magenta), β-strand (blue) that interacts with another β-strand (light blue), and the MYND-type Zinc finger (red). (B) Alignment of the core module of *Drosophila* Trus (*Dm*Trus) to its paralog *Drosophila* Zfrp8 (*Dm*Zfrp8). **(C)** Alignment of the core module of *Drosophila* Trus (*Dm*Trus) with Zebrafish PDCD2L (*Danio rerio* PDCD2L). **(D)** Alignment of the core module of *Drosophila* Trus (*Dm*Trus) with yeast TSR4 (*Sc*TSR4). All structures presented are predicted by AlphaFold (https://alphafold.ebi.ac.uk/), and structural alignment was performed using PyMOL (https://pymol.org/2/).
(TIF)

S8 Fig *ci-GAL4* induced Trus expression in *trus* mutant (A) Representative images of brains of *trus*^*4-15*^*/Dftrus* mutants that were induced Trus expression with *ci-GAL4. ci>Trus* expression rescued cell proliferation detected with anti-PH3 staining in brain. The rescued brain size and structure appeared to be similar to *w*^*1118*^ control. (B) Representative images of wing/haltere/leg discs of *trus*^*4-15*^*/Dftrus* mutants that were induced Trus expression with *ci-GAL4. ci-GAL4* induced Trus expression rescued cell proliferation detected with anti-PH3 staining and size of wing/haltere/leg discs. Density of the PH3 foci appears to be higher in a half of each disc. The size of rescued discs is comparable or larger than *w*^*1118*^ control. 16 larvae were dissected, and all showed the similar rescues. Each image shows the maximum intensity Z-projection. Scale bar: 200μm.
(TIF)

S9 Fig Tap-tagged Trus pull-down from *Drosophila* Kc cells. Tap-tagging reveals stable binding of Trus with Sop/RpS2 (String of pearls) and eEF1α1. Coomassie Blue staining of an SDS-PAGE gel after Tap-tagging with Trus. Three dominant bands are seen: (1) Trus (2) eEF1α1 and (3) Sop. Identifications were made using MALDI-Mass spectrometry. Size marker in the left lane (kDa).
(TIF)

S10 Fig Enlarged lymph gland in *trus*^*1*^*/trus*^*1*^ larva. The primary lobe of the lymph gland is marked with white bracket in each panel. In *trus*^*1*^*/turs*^*1*^ panel, a brain lobe is indicated with white arrow for size comparison. Maximum intensity Z-projections are shown. Scale bar: 200μm.
(TIF)

S1 Table *Drosophila* lines that are used in this study. (DOCX)

S2 Table DNA oligos that are used for production of CRISPR/Cas9 *trus* mutants. (DOCX)

S3 Table Trus and EGFP-Trus expression using *da-GAL4* rescues *trus* mutant. (DOCX)

S1 File Data - developmental timing. (XLSX)

S2 File Data - PH3 puncta and area measurement in brain and wing disc in *trus* mutant. (XLSX)

S3 File Data - *trus*RNAi wing size measurement. (XLSX)
