# Supplementary figures and images for: Drosophila Trus, the orthologue of mammalian PDCD2L, is required for proper cell proliferation, larval developmental timing, and oogenesis
Source: PLoS Genet. 2025 Jun 27;21(6):e1011469. doi: 10.1371/journal.pgen.1011469 (PMC12331172; doi:10.1371/journal.pgen.1011469)

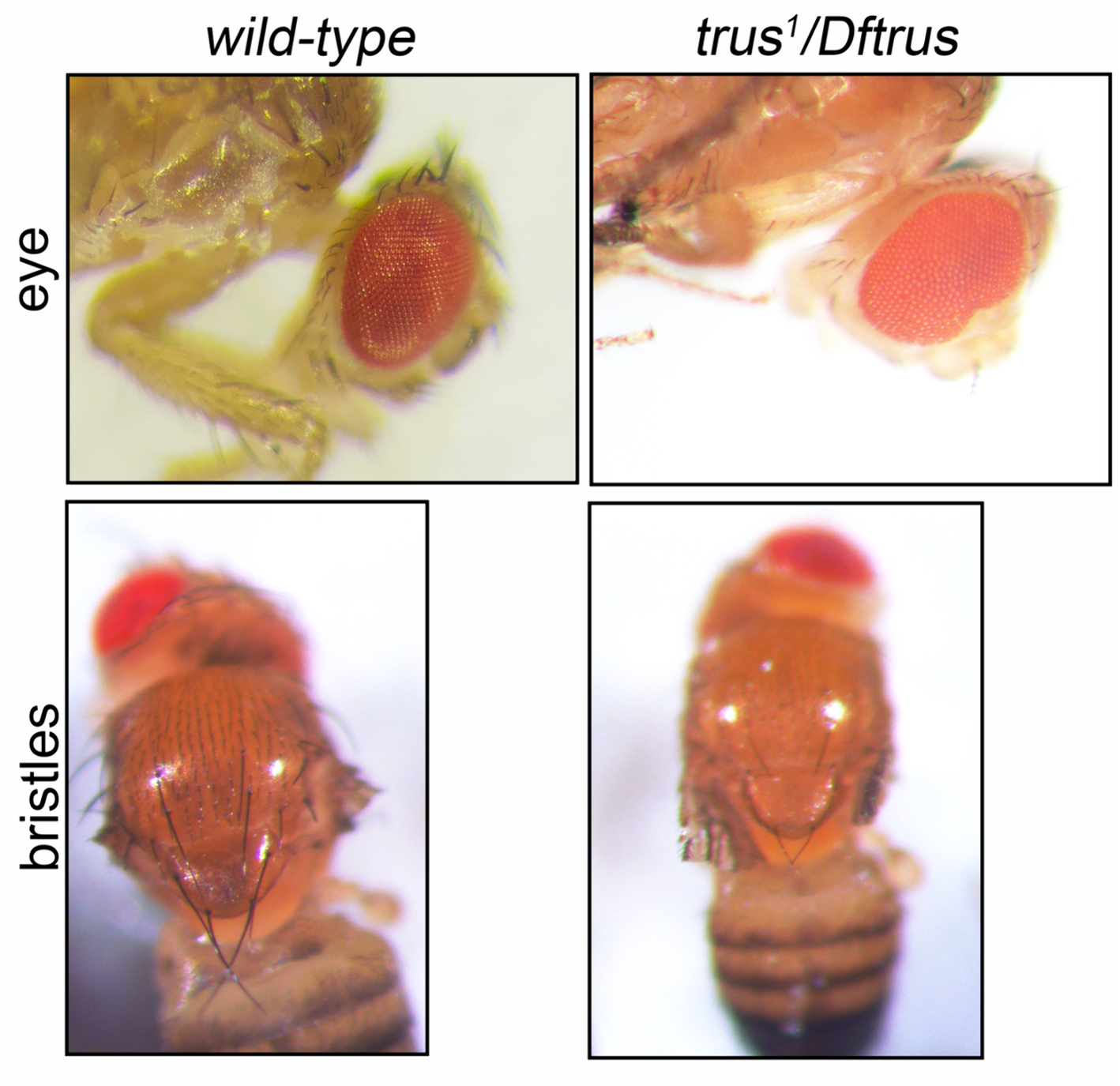

Supplement: S1 Fig — trus1 mutant (trus1/Dftrus) larvae delay development, and most of them are pre-pupal lethal. Rare escaper adults (~1/ vial) eclose showing rough/notched eyes and thin/short bristles which resemble the haplo-insufficiency ‘Minute’ syndrome that is often observed in flies carrying a mutation in one of the genes encoding ribosomal proteins. Wild-type eye and bristles phenotypes are shown in left. (TIF) [file pgen.1011469.s001.tif]

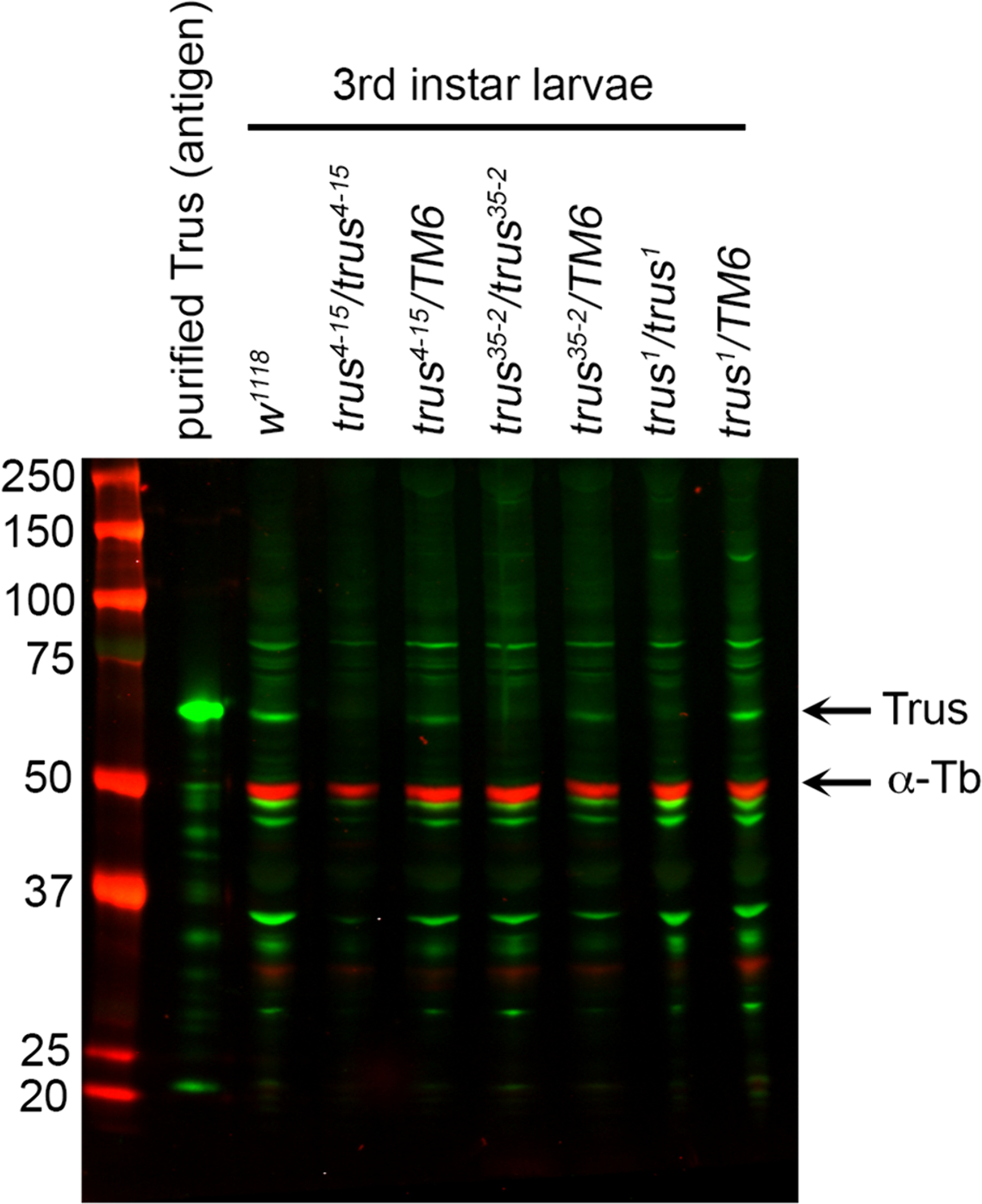

Supplement: S2 Fig — Western blot analysis of third instar larval lysate of trus homozygotes and heterozygotes over a balancer chromosome (TM6B P[Dfd-GMR-nvYPF] Sb). Molecular weight standards are indicated at the left in kDa. Recombinant full-length Trus protein that was purified from Sf9 cells and used as antigen for the anti-Trus antibody production was loaded on lane 2. Larval lysates were loaded from lane 3–9 and genotypes are shown on the top. Green shows signals detected with anti-Trus primary antibody and then DyLight 800-anti-rabbit IgG secondary antibody (Thermo Fisher Scientific). Red shows signals detected with anti-α-Tubulin antibody (DM1A) (Sigma-Aldrich T9026) and DyLight 680-anti-mouse IgG secondary antibody. Full length Trus protein and α-Tubulin (loading control) are indicated by arrows at the right. (TIF) [file pgen.1011469.s002.tif]

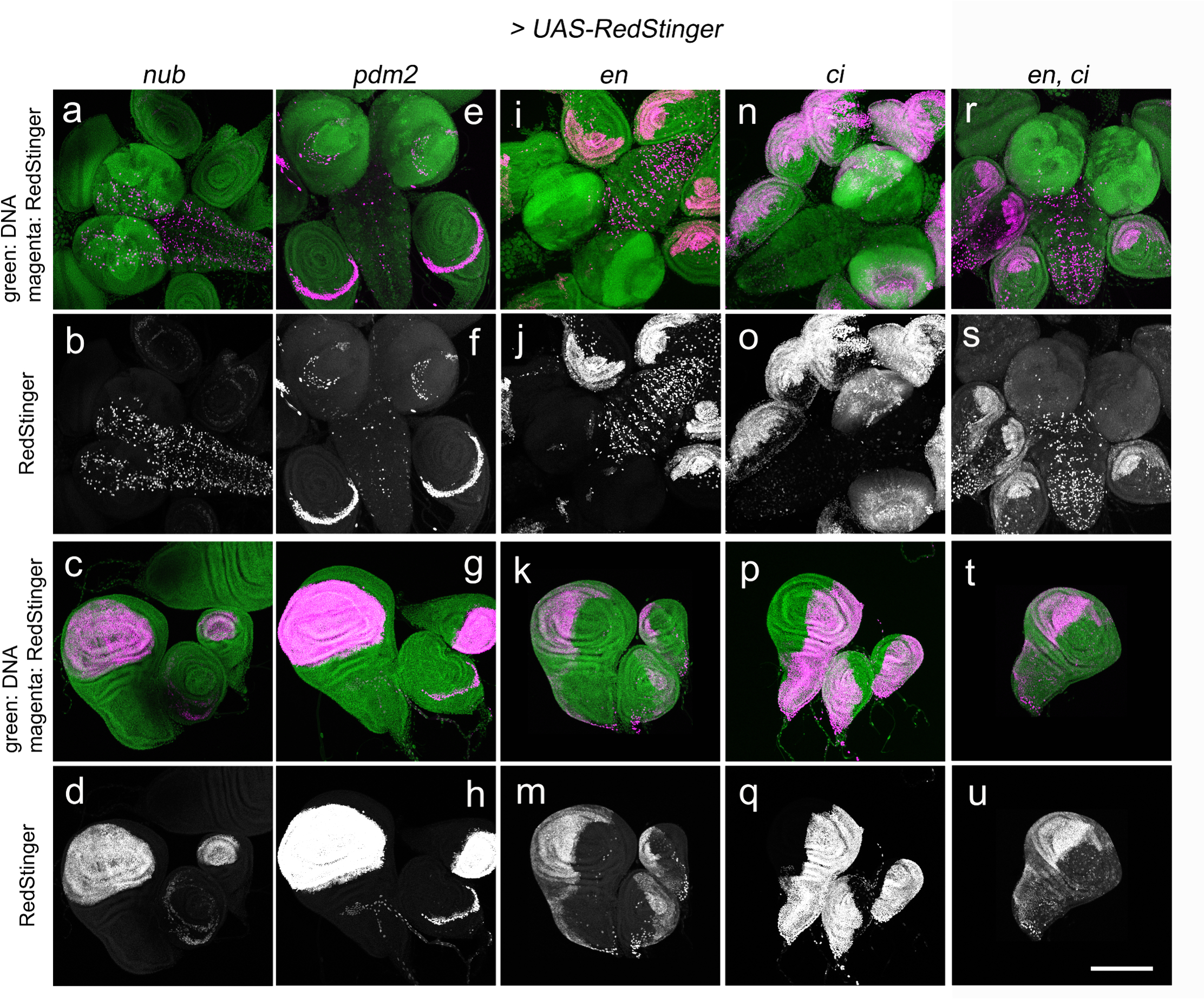

Supplement: S3 Fig — UAS-RedStinger (shown in magenta) was induced with either nub-GAL4 (a-d), pdm2-GAL4 (e-h), en-GAL4 (i-m), ci-GAL4 (n-q), or en-GAL4 plus ci-GAL4 (r-u) drivers. DAPI staining (green) and RedStinger (magenta) are shown in first (a, e, i, n, and r) and third (c, g, k, q, and t) rows. RedStinger (white) is displayed in the second (b, f, j, o, and s) and fourth (d, h, m, q, and u) rows. For each genotype, 10 larvae were dissected, and representative images are shown. Each image shows the maximum intensity Z-projection. Scale bar: 200μm. (TIF) [file pgen.1011469.s003.tif]

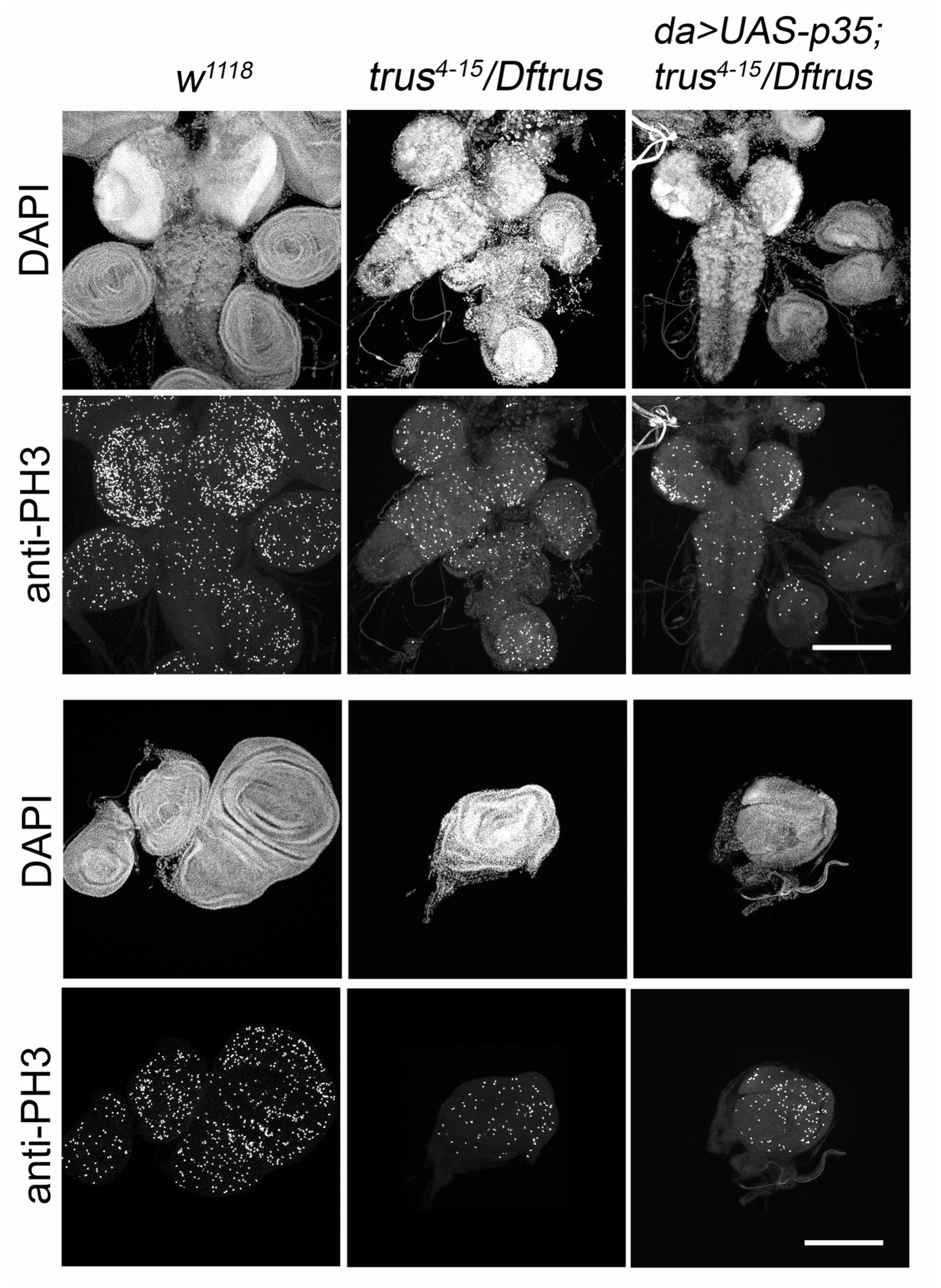

Supplement: S4 Fig — Overexpression of baculovirus p35, an apoptosis inhibitor, did not rescue the defects in tissue growth and cell proliferation in trus mutant larvae. Representative images of brain (top two rows) and wing disc (bottom two rows) from third instar wandering larvae of w1118 (left), trus4-15/Dftrus (middle), and da-GAL4 > UAS-p35 in trus4-15/Dftrus (right) are shown. Fixed tissues were stained with DAPI and anti-PH3 antibody. Brain and wing discs from p35 expressed trus4-15/Dftrus larvae are smaller and show significantly less mitotic cells (PH3 foci) compared to brain and wing discs from w1118 larvae, similar to brain and wing disc from trus4-15/Dftrus larvae without p35 expression, indicating that ubiquitous expression of p35 in trus mutant did not rescue the small brain/wing disc phenotype and cell proliferation defects. 16 larvae of da-GAL4 > UAS-p35; trus4-15/Dftrus were dissected, and all showed the defects similar to trus4-15/Dftrus mutant. Pupariation of p35 expressed trus4-15/Dftrus larvae occurred between 12–15 days AEL similar to trus4-15/Dftrus and the pre-pupae were 100% lethal. Each image shows the maximum intensity Z-projection. Scale bar: 200μm. (TIF) [file pgen.1011469.s004.tif]

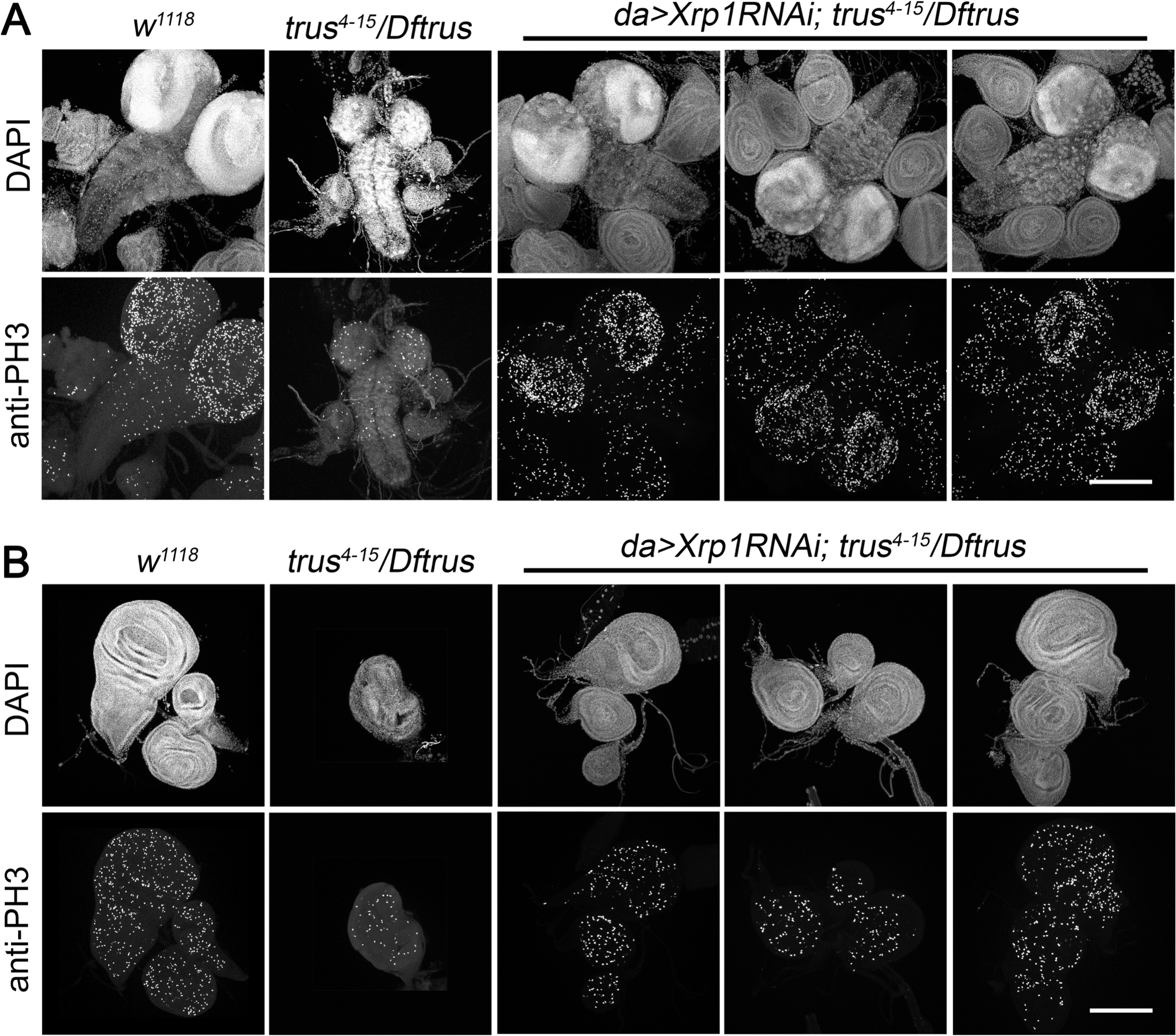

Supplement: S5 Fig — Xrp1 RNAi partially rescues cell proliferation defects in trus mutant brain and wing disc. (A) Representative images of trus4-15/Dftrus mutant brains that were induced Xrp1 RNAi with da-GAL4. Anti-PH3 foci number and brain size in trus4-15/Dftrus mutants were significantly increased by Xrp1 RNAi compared to the trus4-15/Dftrus mutants. (B) Representative images of trus4-15/Dftrus mutant wing/haltere/leg discs that were induced Xrp1 RNAi with da-GAL4. Anti-PH3 foci number and size of wing/haltere/leg discs were moderately increased compared to the trus4-15/Dftrus mutants. 16 larvae were dissected, and representative images are shown. Each image shows the maximum intensity Z-projection. Scale bar: 200μm. (TIF) [file pgen.1011469.s005.tif]

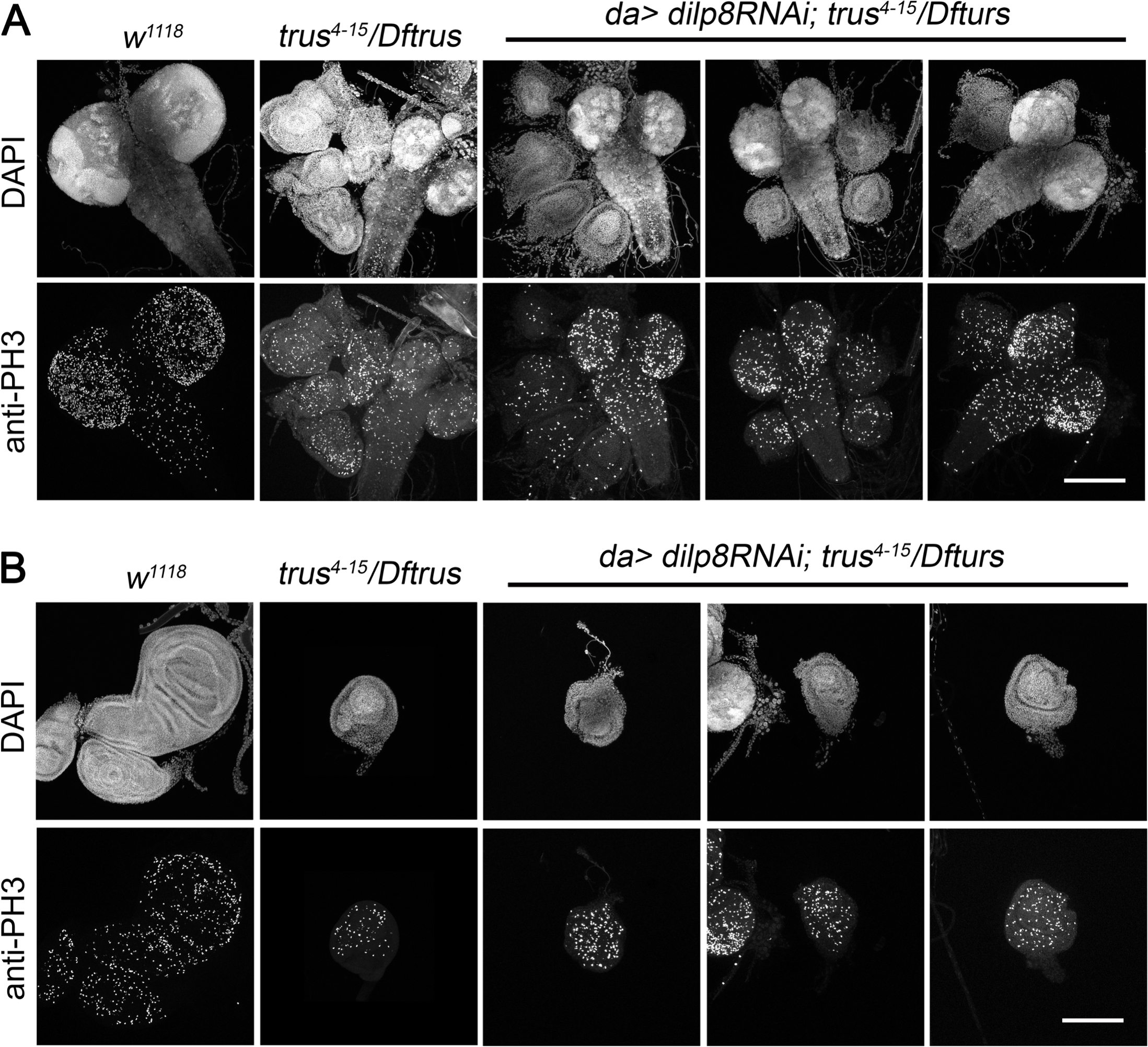

Supplement: S6 Fig — dilp8 RNAi partially rescues cell proliferation defects in trus mutant brain. (A) Representative images of trus4-15/Dftrus mutant brains that were induced Dilp8 RNAi with da-GAL4. Anti-PH3 foci number and brain size in trus4-15/Dftrus mutants were moderately increased compared to the original trus4-15/Dftrus mutants by Dilp8 RNAi, but still less than w1118 level. (B) Representative images of trus4-15/Dftrus mutant wing/haltere/leg discs that were induced Dilp8 RNAi with da-GAL4. Anti-PH3 foci number and size of wing discs was not rescued at all compared to the trus4-15/Dftrus mutants. 16 larvae were dissected, and representative images are shown. Each image shows the maximum intensity Z-projection. Scale bar: 200μm. (TIF) [file pgen.1011469.s006.tif]

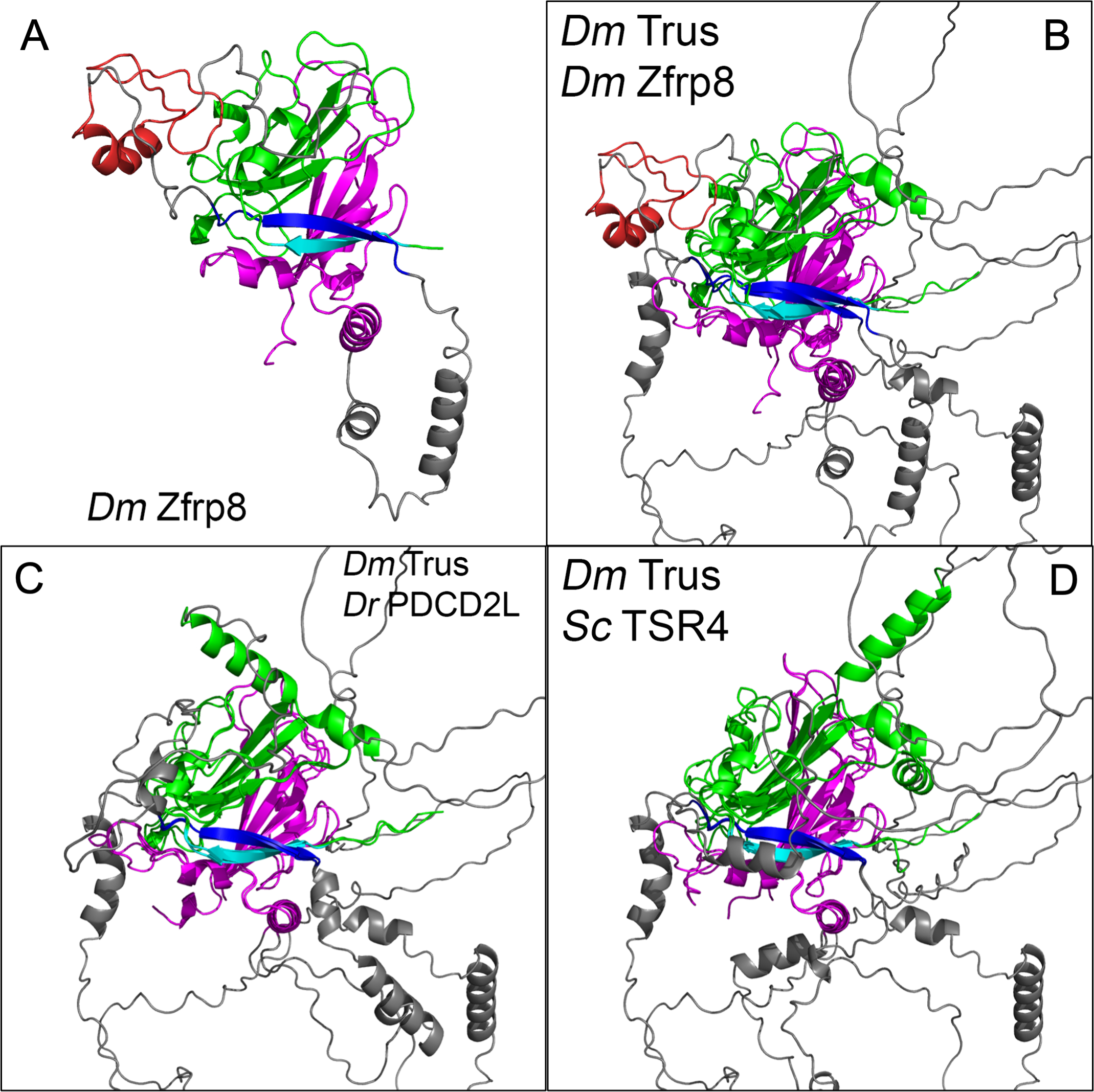

Supplement: S7 Fig — (A) AlphaFold structure of Drosophila melanogaster Zfrp8 (Accession number: Q9W1A3) with domains PDCD2_N (green and light blue), PDCD2_C (magenta), β-strand (blue) that interacts with another β-strand (light blue), and the MYND-type Zinc finger (red). (B) Alignment of the core module of Drosophila Trus (DmTrus) to its paralog Drosophila Zfrp8 (DmZfrp8). (C) Alignment of the core module of Drosophila Trus (DmTrus) with Zebrafish PDCD2L (Danio rerio PDCD2L). (D) Alignment of the core module of Drosophila Trus (DmTrus) with yeast TSR4 (ScTSR4). All structures presented are predicted by AlphaFold (https://alphafold.ebi.ac.uk/), and structural alignment was performed using PyMOL (https://pymol.org/2/). (TIF) [file pgen.1011469.s007.tif]

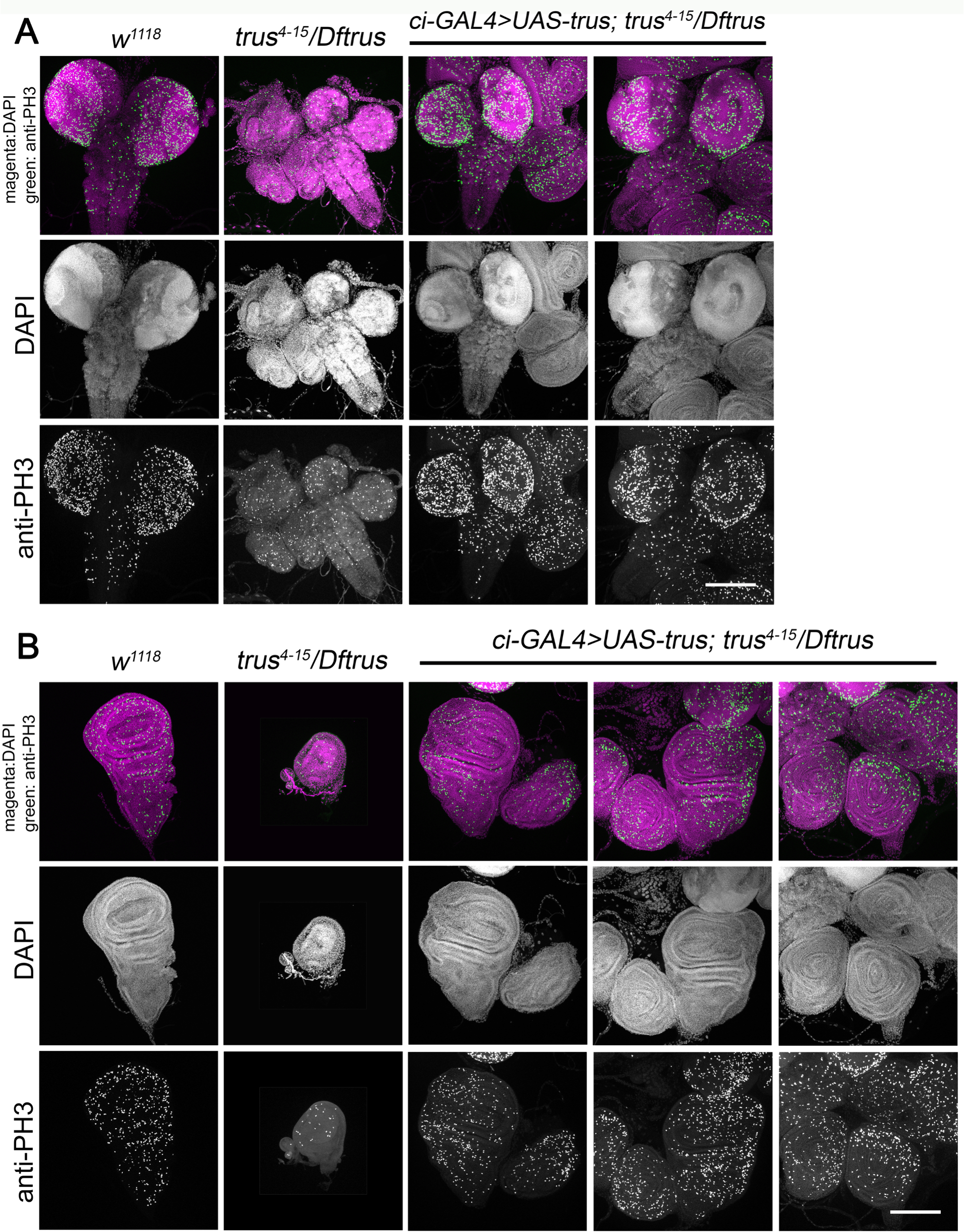

Supplement: S8 Fig — (A) Representative images of brains of trus4-15/Dftrus mutants that were induced Trus expression with ci-GAL4. ci>Trus expression rescued cell proliferation detected with anti-PH3 staining in brain. The rescued brain size and structure appeared to be similar to w1118 control. (B) Representative images of wing/haltere/leg discs of trus4-15/Dftrus mutants that were induced Trus expression with ci-GAL4. ci-GAL4 induced Trus expression rescued cell proliferation detected with anti-PH3 staining and size of wing/haltere/leg discs. Density of the PH3 foci appears to be higher in a half of each disc. The size of rescued discs is comparable or larger than w1118 control. 16 larvae were dissected, and all showed the similar rescues. Each image shows the maximum intensity Z-projection. Scale bar: 200μm. (TIF) [file pgen.1011469.s008.tif]

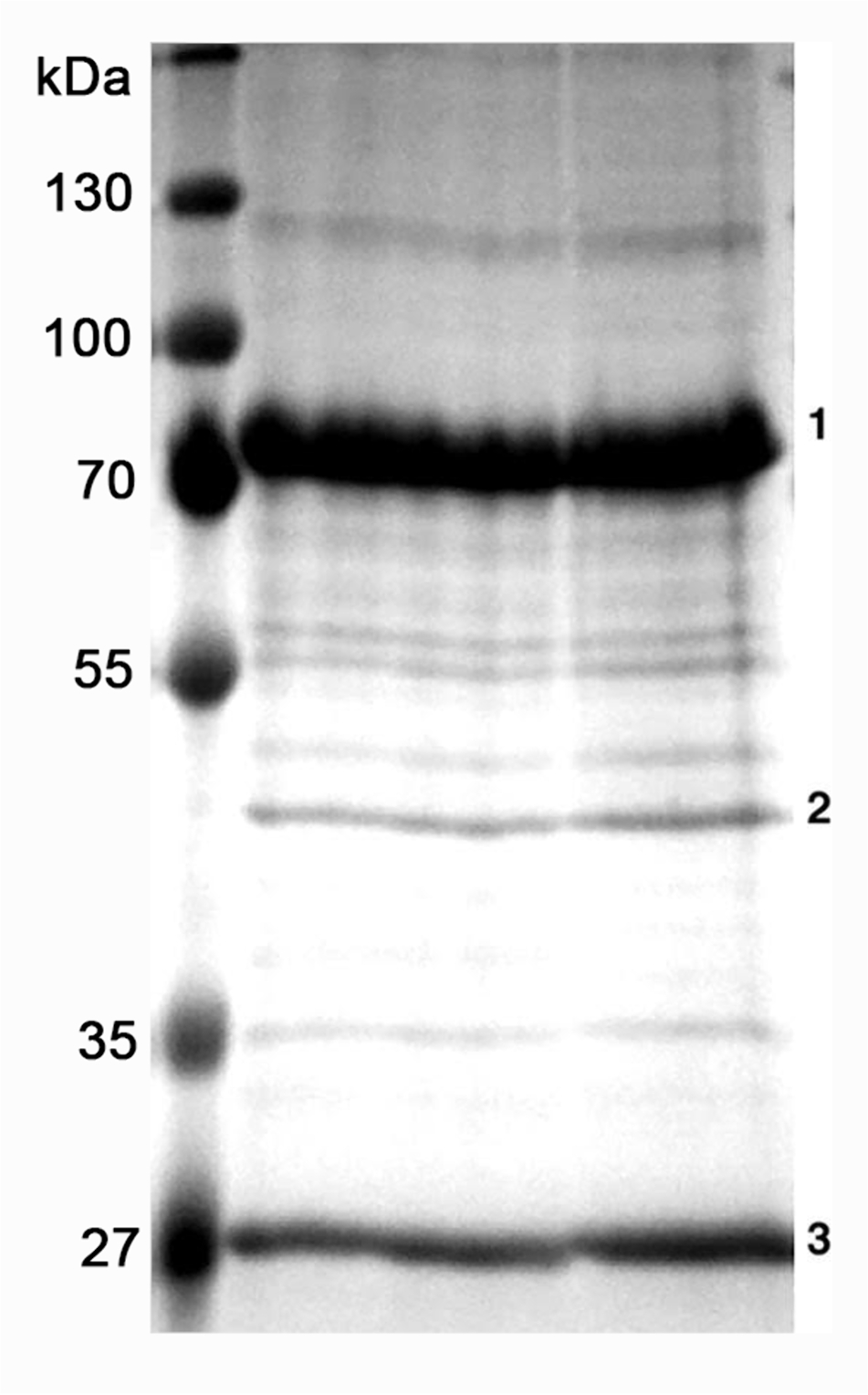

Supplement: S9 Fig — Tap-tagging reveals stable binding of Trus with Sop/RpS2 (String of pearls) and eEF1α1. Coomassie Blue staining of an SDS-PAGE gel after Tap-tagging with Trus. Three dominant bands are seen: (1) Trus (2) eEF1α1 and (3) Sop. Identifications were made using MALDI-Mass spectrometry. Size marker in the left lane (kDa). (TIF) [file pgen.1011469.s009.tif]

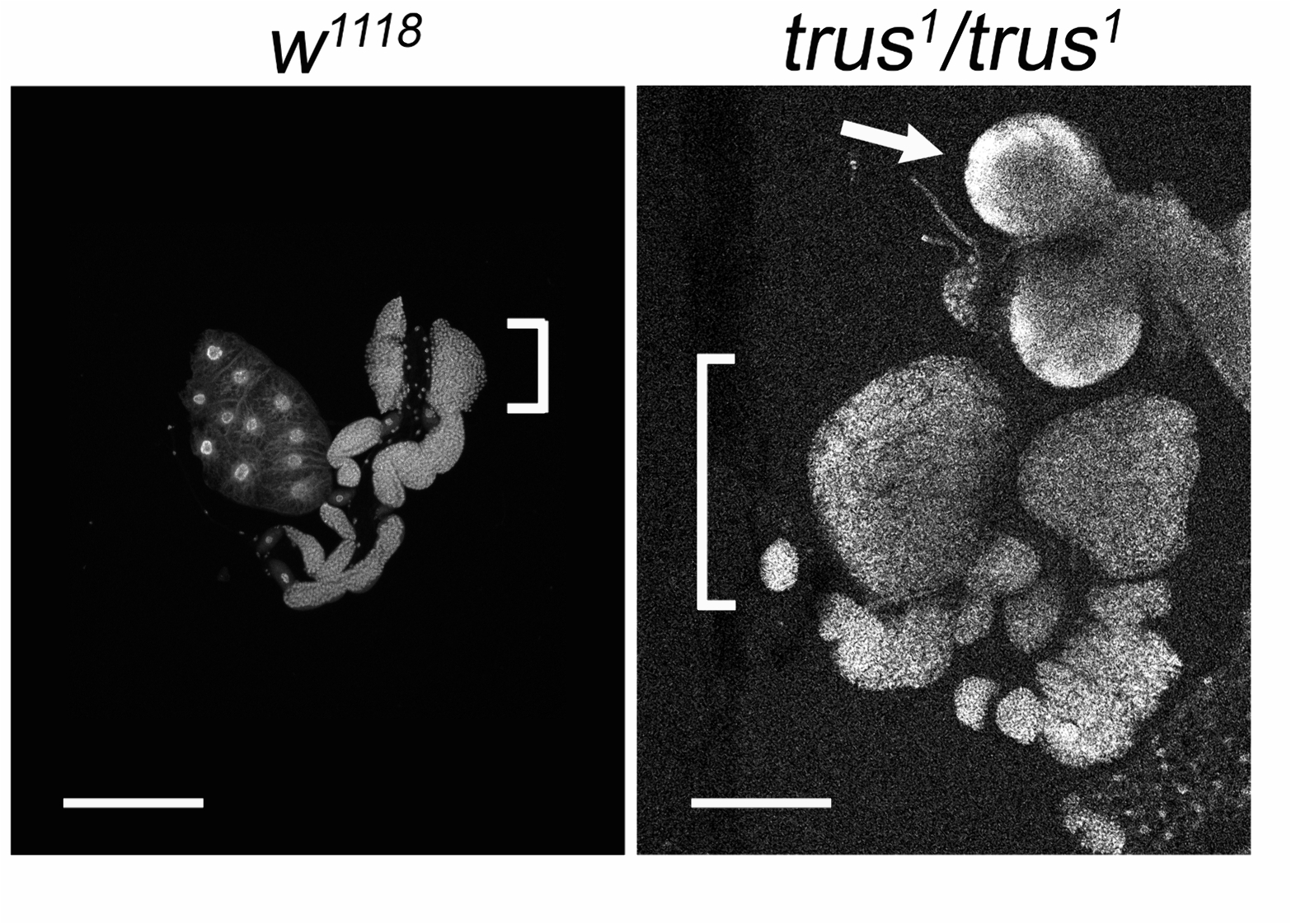

Supplement: S10 Fig — The primary lobe of the lymph gland is marked with white bracket in each panel. In trus1/turs1 panel, a brain lobe is indicated with white arrow for size comparison. Maximum intensity Z-projections are shown. Scale bar: 200μm. (TIF) [file pgen.1011469.s010.tif]
