# Supplementary material for: Drosophila Trus, the orthologue of mammalian PDCD2L, is required for proper cell proliferation, larval developmental timing, and oogenesis
Source: PLoS Genet. 2025 Jun 27;21(6):e1011469. doi: 10.1371/journal.pgen.1011469 (PMC12331172; doi:10.1371/journal.pgen.1011469)
Supplement: S1 Table — (DOCX) [file pgen.1011469.s011.docx]

**S1 Table. *Drosophila* lines that are used in this study.**

| Category | Name used  in this paper | Genotype | Source | Stock number or publication |
| --- | --- | --- | --- | --- |
| Control | *w^1118^* | *w[1118]* | BDSC | BDSC_5905 |
| CRISPR/Cas9 | *vas-Cas9* | *y[1] M{RFP[3xP3.PB] GFP[E.3xP3] =vas-Cas9}ZH-2A w[1118]/FM7c* | BDSC | BDSC_51323 |
| Balancer | *TM6BSbTbHuYFP* | *w[*]; Bl/CyOGFP; TM2/TM6BP[Dfd-GMR-nvYPF]SbTb* | lab stock | Ref.[1] |
| PhiC31 source | *dPhiC3* | *y[1] M{RFP[3xP3.PB] GFP[E.3xP3] =vas-int.Dm}ZH-2A w[*]* | BestGene | BDSC_40161 |
| attP line | VK37 | *y[1] w[1118]; PBac{y[+]-attP-3B}VK00037* | BestGene | BDSC_9752 |
| RNAi | *UAS-trus RNAi* | *w^1118^; P{GD11610}v22067* | VDRC | v22067 |
|  | *UAS-dilpRNAi* | *P{KK112161}VIE-260B* on 2nd | VDRC | v102604 |
|  | *UAS-Xrp1RNAi* | *P{KK104477}VIE-260B* on 2nd | VDRC | v107860 |
|  | *UAS-dicer2* | *w[1118]; ; P{w[+mC]=UAS-Dcr-2.D}10* | BDSC | BDSC_24651 |
| GAL4 drivers | *da-GAL4* | *w[*]; ; P{w[+mW.hs]=GAL4-da.G32}UH1* | lab stock | BDSC_55850 |
|  | *nub-GAL4* | *w[*]; P{w[nub.PK]=nub-GAL4.K}2* | BDSC | BDSC_86108 |
|  | *pdm2-GAL4* | *w[1118]; P{y[+t7.7]w[+mC]=GMR11F02-GAL4}attP2* | BDSC | BDSC_49828 |
|  | *ci-GAL4* | *w[1118]; P{ci-GAL4.C}* | Herman Steller | Ref.[2] |
|  | *en-GAL4* | *y[1] w[*]; P{w[+m*]=GAL4}en[GAL4-33]* | BDSC | BDSC_99568 |
|  | *ci, en-GAL4* | combination of ci-GAL4 and en-GAL4 on 2nd | lab stock |  |
|  | *repo-GAL4* | *w[1118]; P{w[+m*]=GAL4}repo/TM3,Sb[1]* | BDSC | BDSC_7415 |
|  | *spok-GAL4* | *w[1118]; ; P{spok-GAL4,mw+}16A3,17A3* | lab stock | Ref.[3] |
|  | *e22c-GAL4* | *w[*]; P{w[+mW.hs]=en2.4-GAL4}e22c* | BDSC | BDSC_1973 |
|  | *elav-GAL4* | *P{w[+mC]=GAL4-elav.L}2/CyO* | BDSC | BDSC_8765 |
| UAS lines | *dilp8-GFP* | *y[1] w[*]; Mi{y[+mDint2]=MIC}Ilp8[MI00727]* | BDSC | BDSC_33079 |
|  | *UAS-trus* | *w[1118]; P[UAS-Trus,mw+]VK37* | this study |  |
|  | *UAS-EGFP-trus* | *w[1118]; P[UAS-EGFP-Trus,mw+]VK37* | this study |  |
|  | *UAS-p35* | *w[*]; P{w[+mC]=UAS-p35.H}BH1* | BDSC | BDSC_5072 |
|  | *mGFP;RedStinger* | *P{UAS-mGFP}; ; P{w[+mC]=UAS-RedStinger}* | Jae Park |  |
| trus mutants | *trus^4-15^* | *w[1118] ; ; trus[4-15]/TM6B P[Dfd-GMR-nvYPF]SbTb* | this study |  |
|  | *trus^35-2^* | *w[1118] ; ; trus[35-2]/TM6B P[Dfd-GMR-nvYPF]SbTb* | this study |  |
|  | *trus^1^* | *w[1118] ; ; trus[1]/TM6B P[Dfd-GMR-nvYPF]SbTb* | Zucker EMS mutant collection | Ref.[4] |
|  | *Df trus* | *w[1118]; ; Df(3R)BSC847/TM6C, Sb1 cu1* 3R: 12,372,864..12,562,460 | BDSC | BDSC_27920 |
| Zfrp8 mutants | *Zfrp8^P^* | *y[1] w[67c23]; P{lacW}Zfrp8k13705/CyO* | BDSC | BDSC_12199 |
|  | *Df Zfrp8* | *w[1118]; Df(2R)BSC356/SM6a* 2R:24,068,239..24,257,904 (189,666 bp) | BDSC | BDSC_24380 |
|  | *Df(2R)SM206* Zfrp8 null | *Df(2R)SM206 /CyOGFP* | Ruth Steward | Ref.[5] |
|  | *Zfrp8^M-1-1^* | *Zfrp8[M-1-1] /CyOGFP* | Ruth Steward | Ref.[5] |

References

1. Le T, Liang Z, Patel H, Yu MH, Sivasubramaniam G, Slovitt M, et al. A new family of Drosophila balancer chromosomes with a w- dfd-GMR yellow fluorescent protein marker. Genetics. 2006;174(4):2255-7. Epub 20061022. doi: 10.1534/genetics.106.063461. PubMed PMID: 17057238; PubMed Central PMCID: PMCPMC1698648.

2. Croker JA, Ziegenhorn SL, Holmgren RA. Regulation of the Drosophila transcription factor, Cubitus interruptus, by two conserved domains. Dev Biol. 2006;291(2):368-81. Epub 20060118. doi: 10.1016/j.ydbio.2005.12.020. PubMed PMID: 16413529.

3. Shimell M, O'Connor MB. Endoreplication in the. MicroPubl Biol. 2023;2023. Epub 20230221. doi: 10.17912/micropub.biology.000741. PubMed PMID: 36908310; PubMed Central PMCID: PMCPMC9996309.

4. Koundakjian EJ, Cowan DM, Hardy RW, Becker AH. The Zuker collection: a resource for the analysis of autosomal gene function in Drosophila melanogaster. Genetics. 2004;167(1):203-6. doi: 10.1534/genetics.167.1.203. PubMed PMID: 15166147; PubMed Central PMCID: PMCPMC1470872.

5. Minakhina S, Druzhinina M, Steward R. Zfrp8, the Drosophila ortholog of PDCD2, functions in lymph gland development and controls cell proliferation. Development. 2007;134(13):2387-96. Epub 20070523. doi: 10.1242/dev.003616. PubMed PMID: 17522156.
