# Supplementary material for: Drosophila Trus, the orthologue of mammalian PDCD2L, is required for proper cell proliferation, larval developmental timing, and oogenesis
Source: PLoS Genet. 2025 Jun 27;21(6):e1011469. doi: 10.1371/journal.pgen.1011469 (PMC12331172; doi:10.1371/journal.pgen.1011469)
Supplement: S2 Table — (DOCX) [file pgen.1011469.s012.docx]

**S2 Table. DNA oligos that are used for production of CRISPR/Cas9 *trus* mutants.**

| **Name** | **Sequence** | **Use** |
| --- | --- | --- |
| target A sense | 5’ CTTC GGAATGGTCACCTCGTGTCT 3’ | CRISPR mutant |
| target A antisense | 5’ AAAC AGACACGAGGTGACCATTCC 3’ | CRISPR mutant |
|  |  |  |
| target B sense | 5’ CTTC GGATACGATCCCGCTGTTGG 3’ | CRISPR mutant |
| target B antisense | 5’ AAAC CCAACAGCGGGATCGTATCC 3’ | CRISPR mutant |
|  |  |  |
| Trus1 for | 5’ GTACCTAGGATACGAGGATG 3’ | sequencing |
| Trus2 for | 5’ GAAAGACTTGAATGAAACCATG 3’ | PCR |
| Trus3 rev | 5’ CATGACGGAATGGTCACC 3’ | sequencing |
| Trus1 rev | 5’ GTTGCACCAGTCTGCAATC 3’ | PCR and sequencing |
| TrusB for | 5’ CAACCGAACAGGCCAAG 3’ | sequencing |
| TrusC for | 5’ CAGGAGTACAAGCTGAGAG 3’ | sequencing |
