# Supplementary material for: Drosophila Trus, the orthologue of mammalian PDCD2L, is required for proper cell proliferation, larval developmental timing, and oogenesis
Source: PLoS Genet. 2025 Jun 27;21(6):e1011469. doi: 10.1371/journal.pgen.1011469 (PMC12331172; doi:10.1371/journal.pgen.1011469)
Supplement: S3 Table — (DOCX) [file pgen.1011469.s013.docx]

**S3 Table. Trus and EGFP-Trus expression using *da-GAL4* rescues *trus* mutant.**

| *da,trus^4-15^ / TM6TbHu x UAS-Trus; Dftrus/TM6TbHu* | | | |  |  |  |
| --- | --- | --- | --- | --- | --- | --- |
|  |  |  |  |  |  |  |
| AEL | Number of eclosed adults | | | | | |
|  | Tb flies *da>UAS-Trus; trus^4-15^/TM6TbHu*  or *da>UAS-Trus; Dftrus/TM6TbHu* | | | non-Tb flies *da>UAS-Trus; trus^4-15^/Dfturs* | | |
|  | male | female | M+F | male | female | M+F |
| 11 | 10 | 5 | 15 | 18 | 20 | 38 |
| 12 | 7 | 13 | 20 | 9 | 7 | 16 |
| 13 | 5 | 5 | 10 | 2 | 2 | 4 |
| 14 | 8 | 3 | 11 | 0 | 0 | 0 |
| Total | 30 | 26 | 56 | 29 | 29 | 58 |
|  |  |  |  |  |  |  |
|  |  |  |  |  |  |  |
|  |  |  |  |  |  |  |
| *da,trus^4-15^/TM6TbHu x UAS-EGFP-Trus; Dftrus/TM6TbHu* | | | |  |  |  |
|  |  |  |  |  |  |  |
| AEL | Number of eclosed adults | | | | | |
|  | Tb flies *da>UAS-EGFP-Trus; trus^4-15^/TM6TbHu*  or *da>UAS-EGFP-Trus; Dftrus/TM6TbHu* | | | non-Tb flies *da>UAS-EGFP-Trus; trus^4-15^/Dfturs* | | |
|  | male | female | M+F | male | female | M+F |
| 11 | 11 | 7 | 18 | 18 | 21 | 39 |
| 12 | 5 | 4 | 9 | 9 | 7 | 16 |
| 13 | 3 | 1 | 4 | 4 | 2 | 6 |
| 14 | 6 | 2 | 8 | 1 | 0 | 1 |
| Total | 25 | 14 | 39 | 32 | 30 | 62 |
